# Is there a common water-activity limit for the three domains of life?

**DOI:** 10.1038/ismej.2014.219

**Published:** 2014-12-12

**Authors:** Andrew Stevenson, Jonathan A Cray, Jim P Williams, Ricardo Santos, Richa Sahay, Nils Neuenkirchen, Colin D McClure, Irene R Grant, Jonathan DR Houghton, John P Quinn, David J Timson, Satish V Patil, Rekha S Singhal, Josefa Antón, Jan Dijksterhuis, Ailsa D Hocking, Bart Lievens, Drauzio E N Rangel, Mary A Voytek, Nina Gunde-Cimerman, Aharon Oren, Kenneth N Timmis, Terry J McGenity, John E Hallsworth

**Affiliations:** 1 Institute for Global Food Security, School of Biological Sciences, MBC, Queen's University Belfast, Belfast, Northern Ireland, UK; 2 Laboratório de Análises, Instituto Superior Técnico, Lisboa, Portugal; 3 University of Essex, School of Biological Sciences, Colchester, Essex, UK; 4 School of Life Sciences, North Maharashtra University, Jalgaon, Maharashtra, India; 5 Department of Food Engineering and Technology, Institute of Chemical Technology, Mumbai, India; 6 Department of Physiology, Genetics and Microbiology, University of Alicante, Alicante, Spain; 7 CBS Fungal Biodiversity Centre, Utrecht, The Netherlands; 8 CSIRO Food and Nutrition, North Ryde, New South Wales, Australia; 9 Microbial Ecology and Biorational Control, Scientia Terrae Research Institute, Sint-Katelijne-Waver, Belgium; 10 Instituto de Pesquisa e Desenvolvimento, Universidade do Vale do Paraíba, São José dos Campos, São Paulo, Brazil; 11 NASA Headquarters, Washington, DC, USA; 12 Department of Biology, Biotechnical Faculty, University of Ljubljana, Ljubljana, Slovenia; 13 Hebrew University of Jerusalem, Department of Plant and Environmental Sciences, Alexander Silberman Institute of Life Sciences, Jerusalem, Israel; 14 Institute of Microbiology, Technical University Braunschweig, Braunschweig, Germany

## Abstract

Archaea and Bacteria constitute a majority of life systems on Earth but have long been considered inferior to Eukarya in terms of solute tolerance. Whereas the most halophilic prokaryotes are known for an ability to multiply at saturated NaCl (water activity (a_w_) 0.755) some xerophilic fungi can germinate, usually at high-sugar concentrations, at values as low as 0.650–0.605 a_w_. Here, we present evidence that halophilic prokayotes can grow down to water activities of <0.755 for *Halanaerobium lacusrosei* (0.748), *Halobacterium* strain 004.1 (0.728), *Halobacterium* sp. NRC-1 and *Halococcus morrhuae* (0.717), *Haloquadratum walsbyi* (0.709), *Halococcus salifodinae* (0.693), *Halobacterium noricense* (0.687), *Natrinema pallidum* (0.681) and haloarchaeal strains GN-2 and GN-5 (0.635 *a*_w_). Furthermore, extrapolation of growth curves (prone to giving conservative estimates) indicated theoretical minima down to 0.611 *a*_w_ for extreme, obligately halophilic Archaea and Bacteria. These were compared with minima for the most solute-tolerant Bacteria in high-sugar (or other non-saline) media (*Mycobacterium* spp., *Tetragenococcus halophilus*, *Saccharibacter floricola*, *Staphylococcus aureus* and so on) and eukaryotic microbes in saline (*Wallemia* spp., *Basipetospora halophila*, *Dunaliella* spp. and so on) and high-sugar substrates (for example, *Xeromyces bisporus*, *Zygosaccharomyces rouxii*, *Aspergillus* and *Eurotium* spp.). We also manipulated the balance of chaotropic and kosmotropic stressors for the extreme, xerophilic fungi *Aspergillus penicilloides* and *X. bisporus* and, via this approach, their established water-activity limits for mycelial growth (∼0.65) were reduced to 0.640. Furthermore, extrapolations indicated theoretical limits of 0.632 and 0.636 a_w_ for *A. penicilloides* and *X. bisporus*, respectively. Collectively, these findings suggest that there is a common water-activity limit that is determined by physicochemical constraints for the three domains of life.

## Introduction

Water availability (water activity (a_w_)) determines both the vitality and functionality of living systems. The majority of microbes cannot multiply below 0.900 a_w_ (Brown, 1976; Manzoni *et al.*, 2012; Moyano *et al.*, 2013) and for the most extremophilic species, cell division has only been observed down to ∼0.61 a_w_ (Pitt, 1975; Williams and Hallsworth, 2009). The established water-activity window for cell division of archaeal and bacterial life (1–0.755; see Anderson, 1954; Grant, 2004) is narrower than that of some xerophilic fungi that are even able to grow and/or germinate in the range 0.755–0.605 a_w_ (Pitt, 1975; Williams and Hallsworth, 2009). Hence the maxim that eukaryotic systems have evolved levels of solute tolerance superior to those of prokaryotes (Pitt, 1975; Brown, 1976; Williams and Hallsworth, 2009; Rummel *et al.*, 2014).

Microbes are exposed to hostile conditions because of the spatial heterogeneity of their habitats and the temporal dynamics of environmental stress parameters (Lomstein *et al.*, 2012; Cray *et al.*, 2013a; Valentine, 2013; Rummel *et al.*, 2014), as well as collateral damage induced to their macromolecular systems by the solute activities of their own chaotropic and hydrophobic metabolites (Hallsworth, 1998; Bhaganna *et al.,* 2010; Cray *et al.*, 2013a, b, 2014; Ball and Hallsworth, 2014). Recent studies have addressed the means by which temperature, chaotropicity, hydrophobicity, pH and radiation determine the limits of the functional biosphere (for examples see Kashefi and Lovley, 2003; Cowan and Tow, 2004; Hallsworth *et al.*, 2007; Bhaganna *et al.*, 2010; Chin *et al.*, 2010; Golyshina, 2011; Cray *et al.*, 2013b; Harrison *et al.*, 2013; Krisko and Radman, 2013; Yakimov *et al.*, 2014). In contrast, there is a paucity of studies to investigate whether physiological processes can occur in low water-activity environments hitherto considered hostile to biological activity. From cultivation-based studies, the majority of Archaea appear to be extremophilic, whereas Bacteria account for the majority of the biodiversity on Earth (Whitman *et al.*, 1998). It is some members of these domains that hold current records for microbial stress tolerance towards high temperature, chaotropicity, acidity and radiation (Cowan and Tow, 2004; Baker-Austin and Dopson, 2007; Hallsworth *et al.*, 2007; Golyshina, 2011; Cray *et al.*, 2013a; Krisko and Radman, 2013; Yakimov *et al.*, 2014; this article is concerned with the ability to retain metabolic activity and undergo cell division rather than the ability to survive in a dormant condition).

In consequence, we consider it highly unlikely that the cellular biology of these prokaryotes is less capable than that of stress-tolerant members of the Eukarya at high solute concentrations. This study was therefore carried out to determine whether there is a common water-activity limit for the three domains of life; we hypothesised that: (1) halophilic Archaea and Bacteria are capable of cell division below the established 0.755 water-activity limit, that is, this limit is an artefact created by the solubility limit of NaCl rather than a product of their inherent biology; and (2) the most resilient Archaea, Bacteria and Eukarya are equally tolerant to low water-activity.

## Materials and methods

### Organisms and media

**Figure 1:**
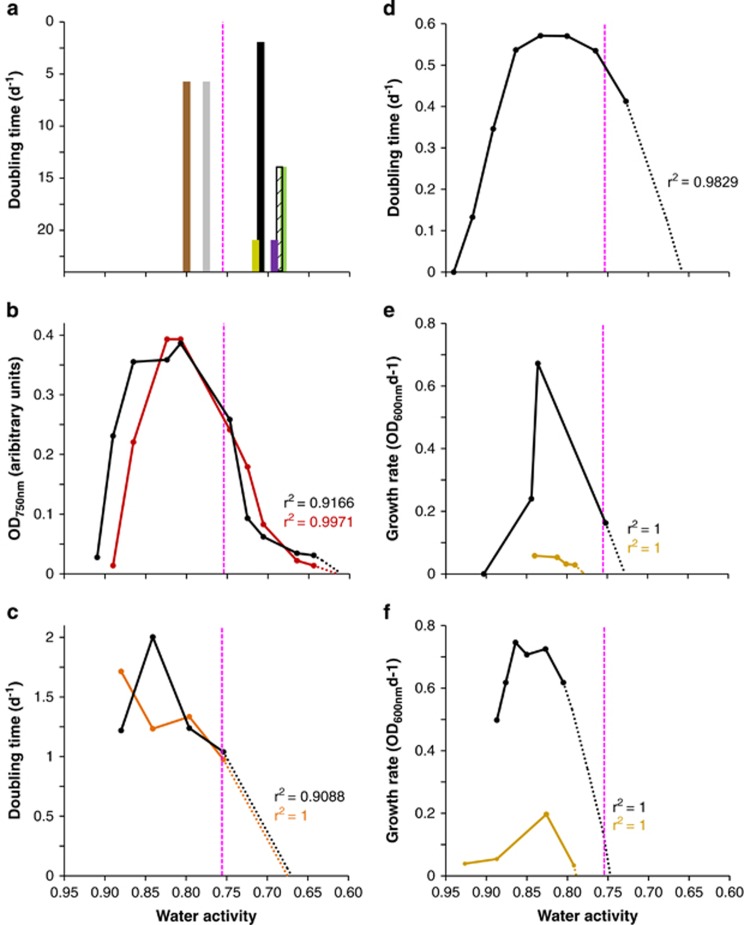
Growth in relation to water activity and/or lower water-activity values at which growth was observed for halophilic Bacteria and Archaea cultured in high-magnesium saline substrates (except for data for *H. saccharovorum* and *Salinicola* strain LC26 that were obtained in high-NaCl media (see Table 1 and Supplementary Table S1), and for *S. ruber* and *S. longa* on NaCl-supplemented media indicated by black lines in (**e**) and (**f**) respectively). (**a**) Halophilic Archaea *Halorubrum saccharovorum* (strain NCIMB 2081^T^; shown in brown), *Halobacterium* sp. NRC-1 and *Halococcus morrhuae* (strain NCIMB 787^T^; both represented in yellow), *Haloquadratum walsbyi* (strain DSM 16790; black), *Halococcus salifodinae* (strain DSM 13046; purple), *Halobacterium noricense* (strain DSM 15987^T^; hashed) and *Natrinema pallidum* (strain NCIMB 777^T^; green), and Bacteria *Salinicola* strain LC26 and *Pontibacillus* strain AS2 (both represented in grey) cultured in media supplemented with various concentrations of NaCl and MgCl_2_ and/or glycerol and ethylene glycol to give a range of water-activity values and incubated at 20 °C for *Pontibacillus* strain AS2 and *Salinicola* strain LC26 or 37 °C for all other species (see Table 1 and Supplementary Table S1). (**b**) Haloarchaeal strains GN-2 and GN-5 shown in orange and black, respectively, cultured in bittern brines supplemented with peptone at 37 °C for 6 days (calculated and replotted against water activity using data from Javor, 1984). (**c**) A mixed halophile community (identified using DAPI; red line) and Bacteria within this community (quantified using molecular probes; black line) by inoculating a synthetic seawater medium with supplemented NaCl using crystalliser brine and incubating at 37 °C (calculated and replotted against water activity using data from Antón *et al.*, 2000). (**d**) The archaeon *Halobacterium* strain 004.1 in a synthetic seawater medium supplemented with NaCl, MgCl_2_, Na_2_SO_4_ and KCl at 37 °C (see Materials and methods and Supplementary Table S2). (**e**) The bacterium *Salinibacter ruber* strain DSM 13855^T^ in complex media supplemented with addition of water from the Dead Sea (0.812 to 0.777 a_w_) and without (0.840 a_w_) at 35 °C (yellow line) or complex media supplemented with NaCl and incubated at 37 °C (black line; calculated and replotted against water activity using data from Sher *et al.*, 2004 and Peña *et al.*, 2010; see also Materials and methods and Supplementary Table S6). (**f**) The bacterium *Salisaeta longa* strain DSM 21114^T^ in complex media supplemented with addition of water from the Dead Sea (0.926 to 0.792 a_w_) at 35 °C (yellow line) or complex media supplemented with NaCl (black line; Materials and methods and Supplementary Table S7). For all media, water-activity values were determined as described in the Materials and methods and at the same temperature as incubation was carried out for each set of media. Curves were extrapolated via regression analyses (dotted lines; for details see Supplementary Table S1) in order to determine the theoretical water-activity minima for growth. Pink dashed lines indicate the previously accepted water-activity limit for extremely halophilic Bacteria and Archaea (see Brown, 1990; Grant, 2004; Kminek *et al.*, 2010).

**Figure 2:**
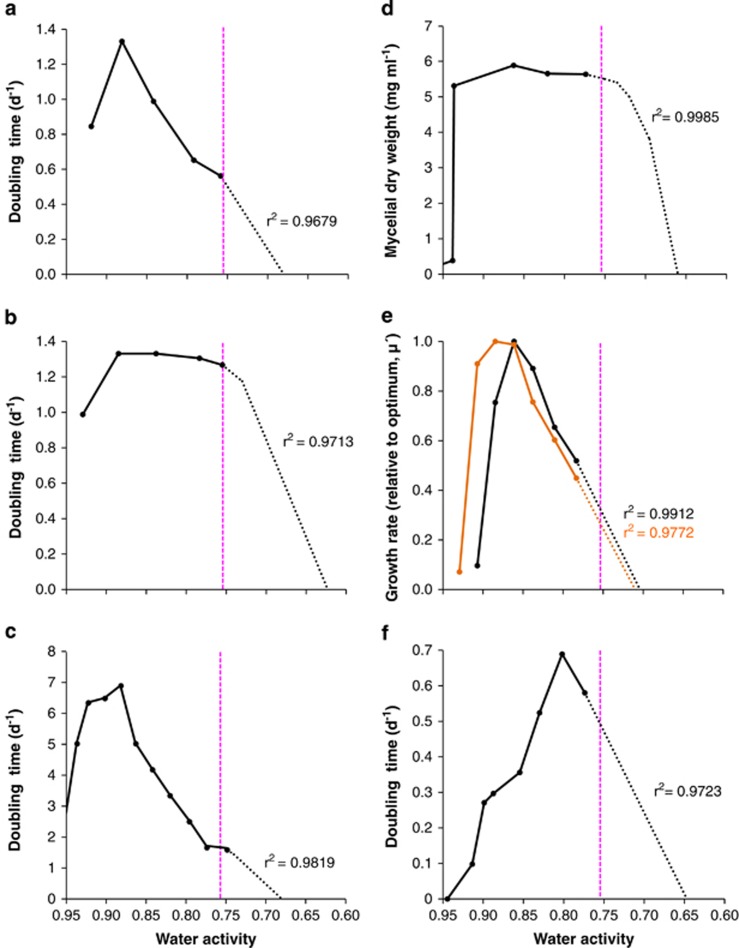
Growth curves for halophilic Bacteria and Archaea cultured in high-NaCl substrates and plotted in relation to water activity. (**a**) The bacterium *Halorhodospira halochloris* (strain and incubation temperature not specified) cultured in a defined medium supplemented with NaCl (calculated and replotted against water activity using data from Deole *et al.*, 2013). (**b**) The bacterium *Halorhodospira halophila* (strain DSM 244^T^; incubation temperature not specified) cultured in a defined medium supplemented with NaCl (calculated and replotted against water activity using data from Deole *et al.*, 2013). (**c**) The bacterium *Halanaerobium lacusrosei* (strain DSM 10165^T^) cultured in a complex medium supplemented with NaCl and incubated at 37 °C (calculated and replotted against water activity using data from Cayol *et al.*, 1995). (**d**) The Aacterium *Actinopolyspora halophila* (strain ATCC 27976^T^) cultured in a complex medium supplemented with NaCl, after a 14-day incubation at 37 °C (calculated and replotted against water activity using data from Yoshida *et al.*, 1991). (**e**) The Archaea ‘Haloarcula californiae' (strain DSM 8905; black line) and ‘Haloarcula sinaiiensis' (strain DSM 8928; orange line) cultured in a complex medium supplemented with NaCl and incubated at 37 °C (calculated and replotted against water activity using data from Javor *et al.*, 1982). (**f**) The archaeon *Halorhabdus utahensis* (strain DSM 12940^T^) in a defined medium supplemented with NaCl and incubated at 30 °C (calculated and re-plotted against water activity using data from Wainø *et al.*, 2000). For all media, water activity values were determined as described in the Materials and methods and at the same temperature as incubation was carried out for each set of media. Curves were extrapolated via regression analyses (dotted lines; for details see Supplementary Table S1) in order to determine the theoretical water activity minima for growth. Pink dashed lines indicate the previously accepted water activity limit for extremely halophilic Bacteria and Archaea (see Brown, 1990; Grant, 2004; Kminek *et al.*, 2010).

**Figure 3:**
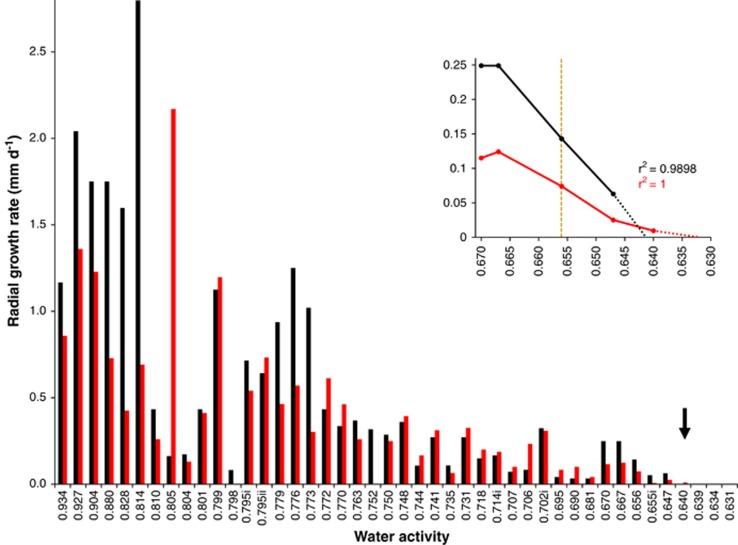
Radial extension rates for two strains of the xerophilic ascomycete *Aspergillus penicillioides* on solid media (MYPiA) supplemented with glycerol and other solutes over a range of concentrations, buffered at various pH values and incubated at different temperatures (Supplementary Table S5) and plotted in relation to water activity: strains JH06THH (black bars) and JH06THJ (red bars). For *A. penicilliodes* strain JH06THH, data relating to the following media were replotted from Williams and Hallsworth (2009): 0.647, 0.656, 0.667 and 0.670 a_w_. Medium composition and incubation temperatures for several treatments with common water-activity values differed (that is, 0.655i, 0.655ii, 0.702i, 0.702ii 0.714i, 0.714ii, 0.795i and 0.795ii; for details see Supplementary Table S8). The black arrow indicates the lowest water-activity at which growth of each strain was observed during an incubation period of six months. The line graph shows extrapolated growth curves plotted using data obtained on the biologically permissive media only in order to determine the theoretical extent of the water-activity windows for growth of each species; the yellow dashed line indicates the original water-activity limit for hyphal growth of the most xerophilic fungi (Pitt and Christian, 1968). For growth rate values of *>*0.75 mm per day, variation was ±0.10 mm per day, and for those of <0.75 mm per day, variation was ±0.040 mm per day (see Williams and Hallsworth, 2009).

**Figure 4:**
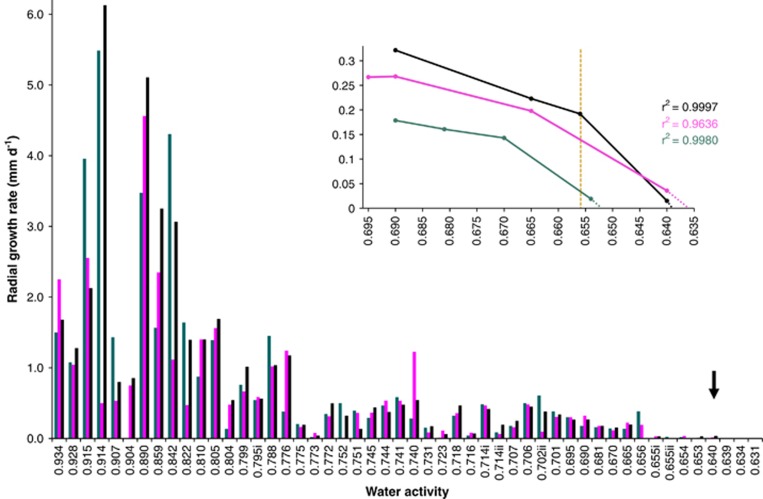
Radial extension rates for three strains of the xerophilic ascomycete *Xeromyces bisporus* on solid media (MYPiA) supplemented with glycerol and other solutes over a range of concentrations, buffered at various pH values and incubated at different temperatures (Supplementary Table S5) and plotted in relation to water activity: strains FRR 0025 (blue bars), FRR 2347 (black bars) and FRR 3443 (pink bars). For all three strains of *X. bisporus*, data relating to the following media were replotted from Williams and Hallsworth (2009): 0.647, 0.653, 0.655ii, 0.656, 0.665, 0.670, 0.702ii and 0.714ii a_w_. Medium composition and incubation temperatures for several treatments with common water-activity values differed (that is, 0.655i, 0.655ii, 0.702i, 0.702ii, 0.714i, 0.714ii, 0.795i and 0.795ii; for details see Supplementary Table S8). The black arrow indicates the lowest water-activity at which growth of each strain was observed during an incubation period of six months. The line graph shows extrapolated growth curves plotted using data obtained on the biologically permissive media only in order to determine the theoretical extent of the water-activity windows for growth of each species; the yellow dashed line indicates the original water-activity limit for hyphal growth of the most xerophilic fungi (Pitt and Christian, 1968). For growth rate values of >4.0 mm per day, variation was ±0.20 mm per day, for those between 0.75 and 4.0 mm per day, variation was ±0.10 mm per day, and for those of <0.75 mm per day, variation was ±0.040 mm per day (see Williams and Hallsworth, 2009).

**Figure 5:**
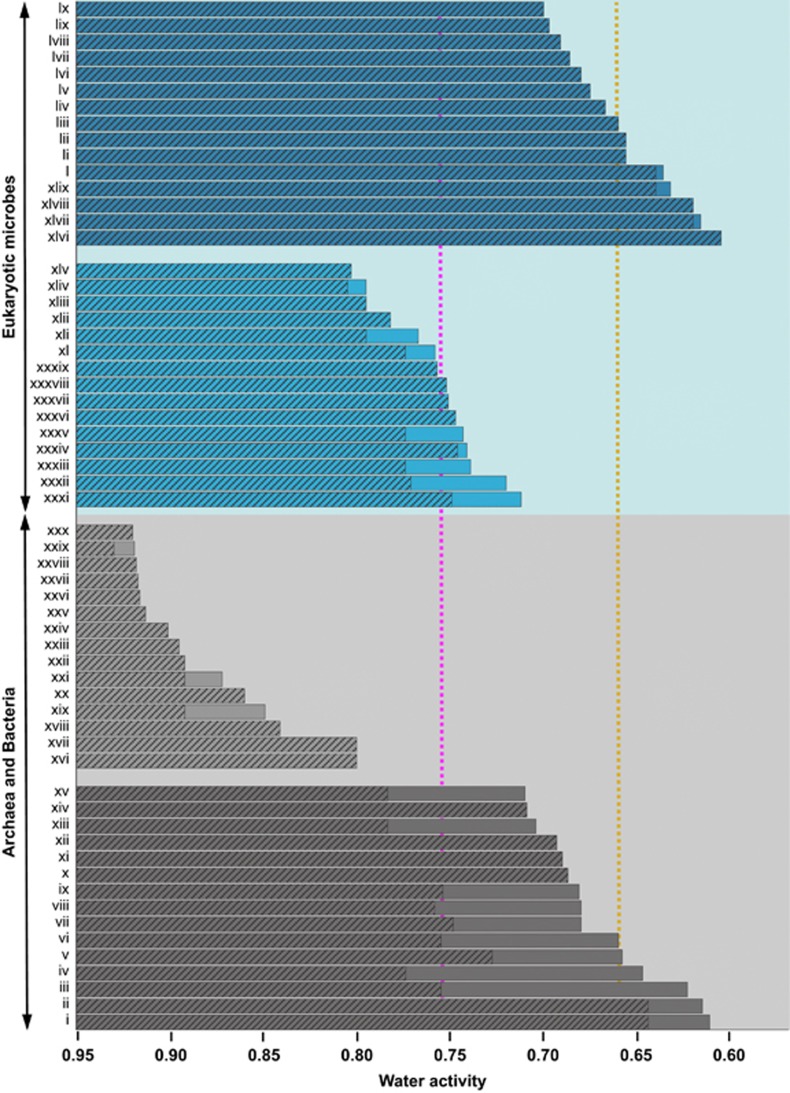
Lower water-activity limits for cell division of the most xerophilic eukaryotic microbes (upper pale-blue panel) and Bacteria and Archaea thus far identified (lower pale-grey panel) on salt-supplemented substrates (mid-blue and dark-grey bars, respectively) and sugar- or polyol-supplemented substrates (dark-blue and mid-grey bars, respectively): (i) haloarchaeal strain GN-5 (Figure 1b), (ii) haloarchaeal strain GN-2 (Figure 1b), (iii) *Halorhodospira halophila* (strain DSM 244^T^; Figure 2b), (iv) *Halorhabdus utahensis* (strain DSM 12940^T^; Figure 2f), (v) *Halobacterium* strain 004.1 (Figure 1d), (vi) *Actinopolyspora halophila* (strain ATCC 27976^T^; Figure 2d), (vii) *Halanaerobium lacusrosei* (strain DSM 10165^T^; Figure 2c), (viii) *Halorhodospira halochloris* (strain not specified; Figure 2a), (ix) Bacteria within a mixed halophile community (Figure 1c), (x) *Natrinema pallidum* (strain NCIMB 777^T^; Figure 1a and Table 1), (xi) *Halobacterium noricense* (strain DSM 15987^T^; Figure 1a and Table 1), (xii) *Halcococcus salifodinae* (strain DSM 13046; Figure 1a and Table 1), (xiii) ‘Haloarcula californiae' (strain DSM 8905; Figure 2e), (xiv) *Haloquadratum walsbyi* (strain DSM 16790; Figure 1a and Table 1), (xv) ‘Haloarcula sinaiiensis' (strain DSM 8928; Figure 2e), [xvi) *Mycobacterium parascrofulaceum* (strain LAIST_NPS017), (xvii) *Mycobacterium smegmatis* (strain ATCC 10143), (xviii) *Tetragenococcus halophilus* (strains T11 and T15), (xix) *Saccharibacter floricola* (strain DSM 15669^T^), (xx) *Staphylococcus aureus* (strains ATCC 6538P, NA and FM1), (xxi) *Asaia bogorensis* (strain JCM 10569^T^), (xxii) *Gluconacetobacter diazotrophicus* (strain DSM 5601^T^), (xxiii) *Streptomyces albidoflavus* (strain JCM 4198^T^), (xxiv) *Staphylococcus epidermidis*, (xxv) *Halotalea alkalilenta* (strain AW-7^T^), (xxvi) *Streptomyces rectiviolaceus* (strain JCM 9092^T^), (xxvii) *Micromonospora grisea* (strain JCM 3182), (xxviii) *Sarcina* sp. (strain 2b), (xxix) *Lactococcus lactis* (strain not specified), (xxx) *Micromonospora* sp. (strain JCM 3050), (see Supplementary Table S3 for (xvi) to (xxx); Stevenson and Hallsworth, 2014 for (xxiii; xxvi; xxvii; xxx)), (xxxi) *Basipetospora halophila* (strain FRR 2787), (xxxii) *Wallemia ichthyophaga* (strain EXF-994), (xxxiii) *Dunaliella salina* (strain UTEX 200), (xxxiv) *Polypaecilum pisce* (strain FRR 2733), (xxxv) *Dunaliella peircei* (strain UTEX 2192), (xxxvi) *Aspergillus penicilliodes* (strain FRR 2612), (xxxvii) germination of *Wallemia sebi* (strain FRR 1473), (xxxviii) *Eurotium halotolerans* (strain EXF-4356), (xxxix) *Halocafeteria seosinensis* (strain EHF34), (xl) *Dunaliella parva* (strain UTEX 1983), (xli) *Pleurostomum flabellatum* (strain CCAP 1959/1), (xlii) *Hortaea werneckii* (strain EXF-225), (xliii) *Euplaesiobystra hypersalinica* (strain CCAP 1528/1), (xliv) *Wallemia muriae* (strain EXF-951), (xlv) *Debaryomyces hansenii* (strain DSM 70590); see Supplementary Table S2 for entries (xxxi) to (xlv), (xlvi) germination of *Xeromyces bisporus* (strain FRR 0025) on a watchglass in a humidity-controlled atmosphere (Pitt and Christian, 1968), (xlvii) *Zygosaccharomyces rouxii* (strain not specified) on a high-sugar medium (von Schelhorn, 1950), (xlviii) germination of *Aspergillus echinulatus* (strain not specified) on a watchglass in a humidity-controlled atmosphere (Snow, 1949), (xlix) *A. penicillioides* (strain JH06THJ) (Figure 3), (l) *X. bisporus* (strain FRR 3443) (Figure 4), (li) *Eurotium amstelodami* (strains FRR 2792 and FRR 0475) on media supplemented with glycerol and other solutes (Williams and Hallsworth, 2009), (lii) *Eurotium chevalieri* strain JH06THI (Williams and Hallsworth, 2009), (liii) *Xerochrysium xerophilium* (formerly *Chyrsosporium xerophilum* Pitt *et al.*, 2013) (strain CBS 153.67^T^) on a medium supplemented with glucose and fructose (Leong *et al.*, 2011), (liv) *Eurotium repens* (strain JH06JPD) on a medium supplemented with glycerol and other solutes (Williams and Hallsworth, 2009), (lv) germination and growth of *Eurotium halophilicum* (strain FRR 2471) on a medium supplemented with glucose and fructose (Andrews and Pitt, 1987), (lvi) germination of *Aspergillus penicillioides* (strain not specified) in complex substrates (Pitt and Hocking, 1977); (lvii) germination of *Bettsia fastidia* (formerly *Chrysosporium fastidium*, Pitt *et al.*, 2013) (strain FRR 77) on a watchglass in a humidity-controlled atmosphere (Pitt and Christian, 1968), (lviii) germination of *W. sebi* (strain FRR 1473) on a medium supplemented with glucose and fructose (Pitt and Hocking, 1977), (lix) hyphal growth of *B. fastidia* (strain FRR 77) (Pitt and Christian, 1968; Williams and Hallsworth, 2009), (lx) germination of *Eurotium rubrum* (strain FRR 0326) (Gock *et al.*, 2003). For each bar, the shaded region extends to the lowest empirically determined water-activity value (see also Supplementary Tables S1–S3 and Figures 1, 2, 3, 4). Only lower water-activity limits for growth are indicated (unless spore germination is indicated); note that some of these species may be unable to grow close to a water activity of 1 (for examples, see Figure 1). The pink dashed line indicates the previously accepted water-activity limit for growth of the most halophilic Bacteria and Archaea (see Brown, 1990; Grant, 2004; Kminek *et al.*, 2010); the yellow dashed line indicates the original water-activity limit for hyphal growth of the most xerophilic fungi (Pitt and Christian, 1968).

A series of experimental, culture-based studies were carried out to determine water-activity limits for Archaea and Bacteria at high-salt concentrations (Figures 1 and 2, Table 1 and Supplementary Table S1), for Eukarya on high-sugar substrates (Figures 3, 4, 5 and Supplementary Table S1) and at high-salt concentrations (Supplementary Tables S1 and S2), and for Bacteria at high-sugar concentrations (Supplementary Tables S1 and S3). Details of microbial strains and culture media are given below; for those microbes where growth rates had been derived previously (see Supplementary Table S1; limits were determined as described in sections on ‘Water-activity measurement' and ‘Determination of water-activity windows for biotic activity').

An initial assessment of water-activity limits for halophilic species of Archaea and Bacteria was carried out on saline media ranging from 0.803 to 0.642 a_w_ (Figure 1a and Supplementary Table S4) using *Halobacterium noricense* (DSM 15987^T^), *Halobacterium* sp. NRC-1 (i.e. *Halobacterium salinarum* ATCC 700922), *Halococcus morrhuae* (NCIMB 787^T^), *Halococcus salifodinae* (DSM 13046), *Halorubrum saccharovorum* (NCIMB 2081^T^) and *Natrinema pallidum* (NCIMB 777^T^). These cultures were obtained from the German Collection of Microorganisms and Cell Cultures (DSMZ, Braunschweig, Germany; *Hbt. noricense* and *Hcc. salifodinae*) and the National Collection of Industrial, Food and Marine Bacteria (NCIMB, Aberdeen, UK; *Hcc. morrhuae* and *Hrr. saccharovorum*). *Hbt*. sp. NRC-1 was provided by Helga Stan-Lotter (University of Salzburg, Austria). The composition for the salt-supplemented culture media used to assay these halophiles are detailed in Supplementary Table S4. Cultures were maintained in their respective media and incubated during these experiments at 37 °C. Data obtained were compared with those for *Haloquadratum walsbyi* (DSM 16790), *Pontibacillus* (strain AS2), *Salinicola* (strain LC26), haloarchaeal strains GN-2 and GN-5, *Halorhodospira halochloris*, *Halorhodospira halophila* strain (DSM 244^T^), *Halanaerobium lacusrosei* (strain DSM 10165^T^), *Actinopolyspora halophila* (strain ATCC 27976^T^), ‘Haloarcula californiae' (strain DSM 8905), ‘Haloarcula sinaiiensis' (strain DSM 8928) and *Halorhabdus utahensis* (strain DSM 12940^T^); see Supplementary Materials and Methods).

Studies of halophilic Archaea and Bacteria capable of growth in MgCl_2_-rich and/or NaCl-rich media (Figures 1b–f and 2) were carried out using pure cultures of the following strains: *Halobacterium* strain 004.1 (for source see Norton *et al.*, 1993), *Salinibacter ruber* strain DSM 13855^T^ and *Salisaeta longa* strain DSM 21114^T^. Cultures of *Halobacterium* strain 004.1 were maintained in the nutrient media on a rotary shaker at 37 °C (for details see Supplementary Table S5; Norton *et al.*, 1993). For studies of MgCl_2_ tolerance, *S. ruber* was maintained on Modified Yeast Extract, Salts Broth (0.840 a_w_) containing (per litre): 1.0 g yeast extract (Beckton, Dickinson and Company, Sparks, MD, USA), 5.0 g KCl, 1.25 g CaCl_2_.2H_2_O, 0.625 g NaBr, 0.25 g NaHCO_3_ plus NaCl (3.34 M), MgSO_4_ (0.101 M) and MgCl_2_ (0.0801 M), and incubated at 35 °C. For studies of both NaCl and MgCl_2_ tolerance, *S. longa* was maintained in (per litre) 2.0 g soluble starch (BDH, Leicester, UK), 1.0 g yeast extract (Difco, Oxford, UK), 1.0 g casamino acids, 5.0 g K_2_SO_4_, 0.1 g CaCl_2_.2H_2_O plus NaCl (1.71 M) and MgCl_2_ (0.246 M), and incubated at 35 **°**C. The composition and water-activity values for culture media used to assay *S. ruber* and *S. longa* are detailed in Supplementary Tables S6 and S7, respectively.

Strains of *Aspergillus penicillioides* (JH06THH and JH06THJ) were isolated previously (Williams and Hallsworth, 2009), and are available from the corresponding author, and strains of *Xeromyces bisporus* (FRR 0025, FRR 2347 and FRR 3443) were obtained from CSIRO Food and Nutrition (FRR collection, North Ryde, NSW, Australia). Cultures were maintained on Malt-Extract, Yeast-Extract Phosphate Agar (MYPiA (per litre): 10 g malt-extract w/v (Oxoid, Oxford, UK), 10 g yeast-extract w/v (Oxoid), 15 g agar w/v (Acros, Bridgewater, NJ, USA), 1.0 g K_2_HPO_4_ w/v)) supplemented with either 6.52 M glycerol (*A. penicillioides*) or 2.2 M sucrose (*X. bisporus*) and incubated in sealed polyethylene bags at 30 °C. The composition and water-activity values for culture media used in stress-tolerance assays of xerophile strains are detailed in Supplementary Table S8.

*Debaryomyces hansenii* strain DSM 70590 was used to determine the lower water-activity limit in high-salt media (Supplementary Table S2). Cultures were maintained in Modified Yeast Extract, Casamino Acid, Tri-Sodium Citrate Broth that contained additional NaCl (3.5 M) and MgCl_2_ (0.399 M); 0.803 a_w_ (see Supplementary Table S4) and incubated at 37 °C.

### Quantitation of growth rates

Exponential-phase cells of *Hbt. noricense*, *Hbt*. sp. NRC-1, *Hcc. morrhuae*, *Hcc. salifodinae*, *Hrr. saccharovorum*, *Nnm. pallidum*, *Pontibacillus* strain AS2 and *Salinicola* strain LC26 were used to inoculate modified Payne's media (250 ml in side-arm flasks; Payne *et al.*, 1960) of varying water activities (see Supplementary Table S4) at a starting cell density equivalent to 0.2 OD_600 nm_. Cultures were incubated at 37 °C and cell density was determined by turbidometric estimation using a nephelometer over a period of two months (two replicates; see Table 1 and Figure 1a). Turbidometric readings were used to construct growth curves from which doubling rates (in days) during exponential growth phase were calculated (Table 1 and Figure 1a).

Exponential-phase cells of *Halobacterium* strain 004.1 (1.6 ml of a preculture, OD_560 nm_=0.5) were used to inoculate media (160 in 250 ml Erlenmeyer flasks) with salt concentrations at increasing multiples of the control medium (see Figure 1d and Supplementary Table S5). Flasks were incubated on a rotary shaker (37 °C; 225 r.p.m.) and cell density monitored turbidimetrically (OD_560 nm_). Cell concentration was determined with a Helber haemocytometer (Hawksley, Sussex, UK) and plotted against the water activity (see below).

Exponential-phase cells of *S. ruber* were used to inoculate complex media (100 in 250 ml Erlenmeyer flasks) supplemented with water from the Dead Sea, Israel (35% w/v total salts; see Figure 1e and Supplementary Table S6) to determine tolerance to MgCl_2_/NaCl mixtures in relation to water activity. Cultures (three replicates) were incubated at 35 °C over 6 days during which OD_600 nm_ was regularly determined. Mean values (OD units per day) derived from exponential-phase measurements were plotted against media water-activity (Figure 1e). Exponential-phase cells of *S. longa* were used to determine tolerance to MgCl_2_/NaCl mixtures (100 in 250 ml Erlenmeyer flasks) in relation to water activity (Supplementary Table S7).

For the xerophilic fungi assayed, agar plugs (4-mm diameter) were taken from the periphery of actively growing cultures of *A. penicillioides* and *X. bisporus* and inoculated onto MYPiA supplemented with a range of stressors (Supplementary Table S8). Petri plates were sealed using Parafilm (Neenah, WI, USA) and plates of the same water activity were incubated in bags of polyethylene that allows gaseous exchange but minimises water loss (Ivanov, 2001; Huang *et al.*, 2002; Hallsworth *et al.*, 2003a, 2003b). Colony diameter was measured daily (in perpendicular directions) and used to calculate rates for radial hyphal extension in mm per day (see Williams and Hallsworth, 2009).

Growth of *Debaryomyces hansenii* (Supplementary Table S2), cultured in Modified Yeast Extract, Casamino Acid, Tri-Sodium Citrate Broth supplemented with NaCl and MgCl_2_ (see Supplementary Table S4), was assessed by via nephelometer (see above) for a period of two months at 37 °C. Turbidometric readings were used to construct growth curves, and doubling times during exponential-growth phase were calculated in order to determine the lower water-activity limit (Supplementary Table S2). For details relating to utilisation of growth-related data from previous studies (Supplementary Table S1), see Supplementary Materials and Methods.

### Water-activity measurement

Water-activity values of media were measured using a Novasina Humidat IC-II water-activity machine (Axair Ltd, Pfäffikon, Switzerland) at the same temperature at which the relevant microbial culture was incubated. The apparatus, fitted with an alcohol-resistant humidity sensor and eVALC alcohol filter that prevents interference by volatiles such as ethanol and glycerol (Hallsworth and Nomura, 1999; Axair Ltd), was calibrated using saturated salt solutions of known water-activity (Winston and Bates, 1960). Water-activity determinations were carried out three times using replicate solutions made up on separate occasions; variation between replicate values was within ±0.002 for determinations in the range 1 to 0.900 a_w_, and within ±0.001 for those within the range 0.900 to 0.600 a_w_ (see below).

Media used for stress-tolerance assays of *Pontibacillus* strain AS2, *Salinicola* strain LC26 and *Hqr. walsbyi* (Table 1 and Supplementary Table S1; Bolhuis *et al.*, 2004; Sass *et al.*, 2008), the halophilic bacterial community (largely *Salinibacter*) from crystalliser pond CR-30 (Braç del Port, Alicante, Spain) (Supplementary Table S6 and Figure 1b; Antón *et al.*, 2000) and *S. ruber* (Supplementary Table S6 and Figure 1f; Sher *et al.*, 2004; Peña *et al.*, 2010) were remade for the current study, and then water-activity determinations carried out as described above. The ionic composition of brines used to culture haloarchaeal strains GN-2 and GN-5 (which was determined to an accuracy level within the low-mM range; Javor, 1984) was used to calculate the water activity of these media (Figure 1a). The values obtained were consistent with the conversion of their brine density (Bé°) to water activity by Javor (1989).

Lower water-activity limits for cell division in some of the most halophilic eukaryotes were determined by plotting extant data sets for cell division at high-salt concentrations in relation to water activity (Supplementary Tables S1 and S2). For culture media of algal species *Dunaliella parva* (UTEX 1983), *Dunaliella peircei* (UTEX 2192) and *Dunaliella salina* (UTEX 200), and the fungi *Eurotium halotolerans* (MZKI A-560), *Wallemia ichthyophaga* (EXF-994), *Wallemia sebi* (FRR 1473) and *Wallemia muriae* (EXF-951; Cifuentes *et al.*, 2001; Butinar *et al.*, 2005; Zalar *et al.*, 2005), the water-activity values that correspond to the limit for cell division were calculated according to the concentrations of NaCl and other components of their respective culture media (Supplementary Table S2) by reference to standard water-activity curves that had been plotted using data points derived at the relevant temperature. Lower water-activity limits for cell division of the nanoflagellate species *Euplaesiobystra hypersalinica* (CCAP 1528/1), *Halocafeteria seosinensis* (EHF34) and *Pleurostomum flabellatum* (CCAP 1959/1) were determined by recreating their respective culture media (see Park *et al.*, 2006, 2007, 2009), followed by empirical determinations of water activity (see below) at their respective incubation temperatures (see Supplementary Table S2).

Water-activity limits for cell division in some of the most xerotolerant Bacteria were determined by plotting extant data sets for cell division at high concentrations of sugar (or other non-ionic solutes) in relation to water activity (Supplementary Tables S1 and S3). Water activity values that corresponded to the cell-division limits for *Tetragenococcus halophilus* (strains T11 and T15) were determined by empirical measurements of water activity (see below) of sucrose solutions (30 °C) that were made up based on the °Bx values from Justé *et al.* (2008a, 2008b). Water-activity limits for *Asaia bogorensis* (JCM 10569^T^), *Gluconacetobacter diazotrophicus* (DSM 5601^T^), *Saccharibacter floricola* (DSM 15669^T^), *Pseudomonas putida* (DSM 6125^h^), *Halotalea alkalilenta* (AW-7^T^) and *Rosenbergiella nectarea* (8N4, LMG 26121, DSM 24150) were determined empirically (see below) using sugar solutions that were remade, and then measured at their appropriate incubation temperatures (Jojima *et al.*, 2004; Ntougias *et al.*, 2007; Cray *et al.,* 2013a; Halpern *et al.*, 2013; Supplementary Table S3).

There are various theoretical and empirical ways in which to determine water-activity values (see, for example, Winston and Bates, 1960; Norrish, 1966; Greenspan, 1977; Ferro Fontán and Chirife, 1981, Brown, 1990; Hallsworth and Nomura, 1999, Caurie, 2005; Yu *et al.*, 2009). For undefined media and/or culture media that have been sterilised, poured into Petri plates and/or stored, water activity cannot be predicted due, in part, to water loss as vapour and hence must be determined empirically. Water-activity values obtained via any available technologies are associated with some degree of variation because of the respective limits of uncertainty and accuracy of the latter (see Yu *et al.*, 2009). Documented water-activity values for saturated salt solutions that can be used for the calibration of instruments are relatively accurate (see Winston and Bates, 1960; Yu *et al.*, 2009). For example, Yu *et al.* (2009) demonstrated overall uncertainties for water-activity measurements of only ±0.0012 to ±0.0018, and accuracy levels of 0.0002 to 0.0005. Some of the new generation of commercially available instruments, such as the Novasina Labmaster series (Axair Ltd), are limited by software that can only be calibrated at a small number of fixed water-activity values and temperature points. The Novasina Labmaster series have been programmed using theoretical water-activity values that were derived using data pooled from a small number of empirical measurements and/or a series of (untested) assumptions (Greenspan, 1977), rather than those collated from a large number of (methodologically diverse) sources—and therefore more accurate—by Winston and Bates (1960).

We took precautions to minimise potential errors that can arise from sampling protocol, instrument resolution and discrimination threshold, inaccuracy of measuring equipment and values for constants or other parameters obtained from external sources and so on (including standard values of saturated salt solutions used for calibration; Brown, 1990; Hallsworth and Nomura, 1999; Yu *et al.*, 2009). An earlier model of water-activity apparatus was used—the Novasina Humidat IC-II (see above)—that allows for manual calibration along continuous scales of water activity and temperature, as used in our earlier studies (see, for example, Hallsworth and Magan 1994a, 1994b, 1994c, 1995, 1996; Kashangura *et al.*, 2006; Hallsworth *et al.*, 2007; Chin *et al.*, 2010; Stevenson and Hallsworth, 2014; Yakimov *et al.*, 2014); filters were fitted to prevent interference from volatiles or particulates, and these were installed taking care to prevent tearing (as described previously, Hallsworth and Nomura, 1999); saturated salt solutions were made up using highest-grade salts and these (along with the medium sample which was contained in a closed sample cup, and the Novasina Humidat IC-II sensor) were left to equilibrate at the temperature at which water-activity determinations were carried out for two to three weeks as described by Winston and Bates (1960); determinations were carried out in a Binder MK53 Environmental Simulation Chamber (Binder GmbH, Tuttlingen, Germany) to minimise temperature variation; measurements were invariably made when the sensor reading was below the water activity of the sample to avoid making the sensor desaturate during a reading; for solid media, Petri plates were poured at a temperature of only 5–7 °C above the medium gel-point (which can differ by up to 10 or 15 °C depending on stressor type and concentration; see Cray *et al.*, 2013b) and lids were immediately placed onto plates to minimise loss of water vapour, avoid condensation and minimise any potential difference between water lost upon pouring between the first and last plates of each batch (for the same reason, agar-containing media were made in small volumes where possible and poured rapidly as practically possible); water-activity readings were taken frequently throughout each sample equilibration period in order to enable the construction of curves that help to establish levels for accuracy of values, consistency and sensor functionality; the Novasina Humidat IC-II sensor was allowed to desaturate immediately after each water-activity measurement to avoid growth of xerophilic fungi on the sensor or sensor housing; and other precautions that are listed in the following section. Uncertainty analyses for water-activity determinations using Aqualab equipment (which is based on dew-point measurements rather than humidity readings) that were carried out by Yu *et al.* (2009) established that equipment errors were less than those stated by the manufacturer (that is, ±0.003). This is consistent with our finding that the variation of our values using the now-obsolete Novasin Humidat IC-II (±0.001 in the range 0.900 to 0.600 a_w_) was less than that claimed by the manufacturer for current Labmaster models that use the same sensor technology (that is, ±0.003).

### Determination of water-activity windows for biotic activity

Assessments of microbial growth rates were made on media over a range of water-activity values in order to facilitate the construction of curves to determine water-activity windows (Supplementary Table S1 and Figures 1, 2, 3; von Schelhorn, 1950; Hallsworth and Magan 1994a, 1994b; Hallsworth *et al.*, 1998; Williams and Hallsworth, 2009). As a precaution, water-activity values of media were checked post-incubation (none of these differed from preinoculation values by more than ±0.002). Growth curves were extrapolated where necessary (Supplementary Tables S1–S3 and Figures 1, 2, 3, 4) to predict the failure point of the window. This has been carried out for data obtained from (1) planktonic cultures of diverse microbes in liquid media (von Schelhorn, 1950; Tienungoon *et al.*, 2000; Ferrer *et al.*, 2003; Cray *et al.*, 2013b); (2) spot-test assays for filamentous Bacteria on solid media (Stevenson and Hallsworth, 2014); and (3) radial extension of mycelia for filamentous fungi on solid media (Hallsworth and Magan, 1999; Rosso and Robinson, 2001; Tassou *et al.*, 2007; Williams and Hallsworth, 2009; Huchet *et al.*, 2013). Some of these studies (Rosso and Robinson, 2001; Tassou *et al.*, 2007; Huchet *et al.*, 2013) demonstrated/validated theoretical determinations for water-activity minima with an accuracy consistent with the level of sensitivity of the microbial cell; that is, ±0.002 (Stevenson *et al.*, 2014). However, where growth curves continue in an asymptotic manner, extrapolation may give a conservative estimate for water-activity minima for both fungi (see Figures 1b, 3 and 4) and planktonic cultures of prokaryotic cells (Neumeyer *et al.*, 1997). For studies of temperature, modelling of microbial growth curves can also result in conservative predictions of the actual limits (Tienungoon *et al.*, 2000). In the current study, linear, polynomial or Gausian regression analyses were carried out to predict water-activity minima; for each data set, the regression analysis that gave the highest regression coefficient was employed (Supplementary Table S1). In order to compare water-activity limits for the most resilient microbes known, we selected the 30 eukaryotic microbes (see Supplementary Tables S1 and S2 and Figures 3 and 4) and 30 Archaea or Bacteria (see Supplementary Tables S1 and S3 and Figures 1 and 2) with the highest recorded tolerance to ionic or non-ionic solutes (Figure 5; Stevenson and Hallsworth, 2014; Stevenson *et al.*, 2014). These data were compiled to determine only the lower water-activity limits of microbial growth windows, and were based on biotic activity including germination rates and growth rates, as there are no data sets derived using culture-independent techniques that reveal metabolic activity at such low water-activity values to our knowledge.

## Results and discussion

### A water-activity limit of ∼0.611 for growth of archaeal and bacterial halophiles

A number of halophilic Archaea and Bacteria are not only capable of cell division in salt-saturated substrates, but can have substantial growth rates under these conditions (Javor, 1984; Brown, 1990; Yoshida *et al.*, 1991; Cayol *et al.*, 1995; Grant, 2004; Deole *et al.*, 2013). NaCl is soluble to ∼5.2 M, which is equivalent to 0.755 a_w_ at temperatures in the range of 25–35 °C (the water activity of saturated solutions of NaCl ranges from 0.765 to 0.745 between 2 °C and 50 °C; Winston and Bates, 1960). We selected nine extremely halophilic species of Archaea and Bacteria that belong to a range of phyla, and determined whether they would grow in liquid media supplemented with various stressors to obtain a range of water-activity values from 0.803 to 0.642 (Figure 1a, Table 1 and Supplementary Table S4). At low water-activity values, cell-replication times for the vast majority of halophiles become exponentially long (Brown, 1990). Remarkably, however, the majority of the empirically determined doubling times for these microbes ranged from <7 to ∼21 days at water-activity values of ≤0.717, and four of the selected species grew at even lower water-activity values: 0.709 for *Hqr. walsbyi*, 0.693 for *Hcc. salifodinae*, 0.687 for *Hbt. noricense* and 0.681 for *Nnm. pallidum* (Figure 1a and Table 1). The relatively high growth rates of *Hqr. walsbyi*, *Hbt. noricense* and *Nnm. pallidum*, despite this low water-activity range (0.709 to 0.681), suggest that their actual water-activity minima are <0.681 (likely in the range 0.650 to 0.605; Figure 1a). For some of the media assayed, the NaCl concentrations were relatively low (between 3.3 and 1.5 M) and MgCl_2_ concentrations were relatively high; two of the media contained ethylene glycol or glycerol (Figure 1a, Table 1 and Supplementary Table S4). When compared on a w/v basis, MgCl_2_ depresses water activity more effectively than NaCl (Winston and Bates, 1960; Hallsworth *et al.*, 2007); it is, nevertheless, the potent chaotropicity-mediated stress induced by MgCl_2_ that typically limits microbial systems which are exposed to this salt (Hallsworth *et al.*, 2007; Bhaganna *et al.*, 2010; Cray *et al.*, 2013b; Yakimov *et al.*, 2014). It is for this reason that we substituted MgCl_2_ with ethylene glycol and glycerol for some treatments. Glycerol can protect against chaotrope-induced stresses in phylogenetically diverse microbes (Hallsworth *et al.*, 2003a, 2007; Williams and Hallsworth, 2009; Bhaganna *et al.*, 2010; Bell *et al.*, 2013). This polyol can, however, itself act as a chaotropic stressor at molar concentrations (Williams and Hallsworth, 2009; Cray *et al.*, 2013a). For *Hbt. noricense* and *Hcc. salifodinae*, the partial substitution of salts by either ethylene glycol or glycerol facilitated growth at lower water-activity values than occurred on media with only salts added (Table 1 and Supplementary Table S4). Given the ability of MgCl_2_ to reduce water activity to <0.755 (several studies suggest that MgCl_2_ concentrations in the range 2–3 M can be biologically permissive for some halophiles (Hallsworth *et al.*, 2007; Oren, 2013; Yakimov *et al.*, 2014)), we sought evidence that halophilic Archaea and Bacteria can be active at water activities <0.681 in magnesium-rich media or substrates including bitterns, that is, crystalliser ponds that have naturally elevated magnesium concentrations (Figures 1b–f).

Two strains of haloarchaea that originated from Mexican solar salterns (GN-2 and GN-5) were previously cultured in bittern water (Javor, 1984). We determined the water activity of this substrate (see Materials and methods) and plotted growth data for these strains against water activity (Figure 1b), indicating that although optimal growth was observed between 0.800 and 0.845 a_w_, growth rates were remarkably high at 0.755 a_w_; that is, 61% and 67% of that of the optimum for GN-2 and GN-5, respectively. Despite the short incubation time (6 days), both strains were active even in the most high-salt medium (equivalent to a brine density of 32 Bé° Javor, 1984) at a water activity of 0.635 (Figure 1b). In common with other studies of water-activity and temperature windows for microbial growth (or those for other stress parameters; see Materials and methods), we carried out regression analyses to obtain theoretical water-activity minima for strains GN-2 and GN-5; these were 0.615 and 0.611, respectively (Supplementary Table S1 and Figure 1b). This represents a substantial increase in relation to the previously accepted water-activity window for cell division of archaeal or bacterial halophiles.

We also sought evidence of microbial activity at sub-0.755 water-activity values for halophilic Bacteria that are known to inhabit bittern brines. Water from a crystalliser pond (Pond CR-30, Braç del Port) was previously used to inoculate a synthetic seawater medium supplemented with NaCl, which was then monitored over time by quantifying total cell counts and using molecular probes to identify halophilic Bacteria belonging to the *Salinibacter* assemblage within the community (Antón *et al.*, 2000; Figure 1c). We determined the water activity of these media (see Materials and methods) and plotted these values against growth-rate data for this Bacterial assemblage (Figure 1c). Growth rates were optimal at 0.841 a_w_ (doubling time=12 h) and reduced by only 50% at 0.755 a_w_. Extrapolation of this curve suggests an actual water activity limit in the range 0.675–0.670 (Supplementary Table S1 and Figure 1c). These values were derived from a combined data set representing a number of bacterial populations, and hence it is likely that one or more populations and species is/are capable of growth at lower water activities than this extrapolation suggests.

The biotic windows were also determined for individual Archaea and Bacteria found in magnesium-rich habitats and/or known to have a degree of tolerance towards MgCl_2_ (strains of *Halobacterium*, *Salinibacter* and *Salisaeta;* Figures 1d–f). *Halobacterium* strain 004.1, isolated from a brine pool within a subsurface salt deposit (UK, Norton *et al.*, 1993), shares traits in common with strains of *Hbt. noricense* (McGenity *et al.* 2000), a species that is frequently found in crystals of buried halite (Gramain *et al.*, 2011). This strain remained highly active at 0.728 a_w_ (∼70% of the optimum growth rate) and extrapolation of the curve suggested a lower theoretical limit of 0.658 a_w_ (Supplementary Table S1 and Figure 1d). *S. ruber* can form up to 27% of halophile communities in high-magnesium habitats such as crystalliser ponds in solar salterns (Antón *et al.*, 2000). This bacterium has extraordinary adaptations that enable it to dominate high-salt habitats, including light-activated protein pumps that generate proton-motive force and thereby boost energy generation, use of ions—rather than organic compounds—for osmotic adjustment and cytosolic proteins with physicochemical properties that enable function at high ionic strength (Oren *et al.*, 2002; Balashov *et al.*, 2005; Cray *et al.*, 2013a; Oren and Hallsworth, 2014). *S. longa*, a close relative of *Salinibacter* spp. (Bodaker *et al.*, 2010; Oren, 2013), is also found in hypersaline marine habitats (Vaisman and Oren, 2009). However, we found that high-magnesium media (supplemented with water from the Dead Sea; Supplementary Tables S6 and S7) were not biologically permissive for *S. ruber* and *S. longa* in as much as their growth windows did not extend below 0.755 (Figures 1e and f).

Given that the chaotropicity of MgCl_2_ may have curtailed the growth of *S. ruber* and *S. longa* (Figures 1e and f), we also determined growth rates for these strains in high-NaCl media (Figure 2). Bacteria notorious for obligate extreme halophilicity at high NaCl concentrations have been studied previously: *Actinopolyspora halophila*, *Halanaerobium lacusrosei* (formerly *Haloanaerobium lacusroseus*), *Halorhodospira halochloris* (formerly *Ectothiorhodospira halochloris*) and *Halorhodospira halophila* (formerly *Ectothiorhodospira halophila*; Yoshida *et al.*, 1991; Ollivier *et al.*, 1994; Ruan *et al.*, 1994; Cayol *et al.*, 1995; Imhoff and Süling, 1996; Oren, 2000; Deole *et al.*, 2013), as well as the exceptionally halophilic Archaea ‘Haloarcula californiae', ‘Haloarcula sinaiiensis' and *Halorhabdus utahensis* (Javor *et al.*, 1982; Wainø *et al.*, 2000). We therefore quantified the water-activity limits for each species based on previous empirical growth determinations (Figure 2 and Supplementary Table S1). Two of these species, *A. halophila* and *Hlr. halochloris*, exhibited exceptionally high biomass/growth rates at 0.757 and 0.774 a_w_, respectively, in salt-saturated media that were ∼95% of those corresponding to their water-activity optima (Figures 2b and d, respectively). Unlike *S. ruber, A. halophila* utilizes organic compounds for osmotic adjustment and is known to accumulate high levels of compatible solutes such as ectoine and trehalose (Kar *et al.*, 2014). Extrapolations of growth curves (Supplementary Table S1) indicated theoretical limits of 0.747 for *S. longa* (Figure 1f), 0.725 for *S. ruber* (Figure 1e), 0.710 for ‘Har. sinaiiensis' (Figure 2e), 0.704 for ‘Har. californiae' (Figure 2e), 0.680 for *Hlr. halochloris* (Figure 2a), 0.668 for *H. lacusrosei* (Figure 2c), 0.660 for *A. halophila* (Figure 2d), 0.647 for *Hrd. utahensis* (Figure 2f) and 0.623 for *Hlr. halophila* (Figure 2b). The data presented in Figures 1 and 2 are consistent with the high growth rates of other archaeal and bacterial species known to be extreme obligate halophiles (for example, *Actinopolyspora iraqiensis* (syn. *Saccharomonospora halophila*) strain IQ H2, Ruan *et al.*, 1994; Tang *et al.*, 2011). Furthermore, the majority of the halophilic prokaryotes represented in Figures 1 and 2 achieved optimum growth rates in the range 0.845 to 0.765 a_w_. This water-activity range is lower than, or equivalent to, that of the most xerophilic fungi thus far reported (Pitt, 1975; Brown, 1990; Williams and Hallsworth, 2009), with the exception of *Xeromyces bisporus* (see below).

### Revised lower limit of 0.632 a_w_ for hyphal growth of fungal xerophiles

Before our recent studies involving manipulation of the chao-/kosmotropicity of culture media (Williams and Hallsworth, 2009), the established water-activity limit for mycelial growth in xerophilic fungi was 0.656 (for *X. bisporus*, Pitt and Christian, 1968). In the current article, we attempted to extend biotic windows of xerophilic fungi via manipulations of medium composition and other environmental conditions, and in this way to emulate the study of halophilic prokaryotes described above. Determinations were made for water-activity windows for hyphal extension (rather than germination) to enable comparisons with the stress tolerance of the Archaea and Bacteria (Table 1 and Figures 1 and 2). Furthermore, vegetative growth of mycelia is qualitatively distinct from spore germination in terms of cellular ultrastructure and physiology, growth kinetics and water-activity limits (Ayerst, 1969; Pirt, 1975; Hallsworth and Magan, 1994a, 1995; Leong *et al.*, 2011). Whereas fungal propagules are renowned for their inherent robustness (for example, during desiccation and at extreme pressures and temperatures), this tenacity is only observed during dormancy (Potts, 1994; Chin *et al.*, 2010; Wyatt *et al.*, 2014). For this study, we selected five strains of extremely xerophilic fungi: *X. bisporus* FRR 0025 (reported to grow down to 0.656 and germination down to 0.605 a_w_; Pitt and Christian, 1968); *X. bisporus* strains FRR 2347 and FRR 3443 that are capable of hyphal growth at water activities comparable to strain FRR 0025 (Williams and Hallsworth, 2009); and *Aspergillus penicilliodes* strains JH06THH and JH06THJ that have been reported to be more xerophilic than *X. bisporus* (the former were capable of mycelial growth down to 0.647 a_w_; Williams and Hallsworth, 2009).

The water activity for optimum growth of *A. penicillioides* was found to be between 0.800 and 0.820 (Figure 3); lower than that for *X. bisporus*, other species of xerophilic fungi (Figure 4; Williams and Hallsworth, 2009) and most halophilic Archaea and Bacteria (Figures 1 and 2). Radial growth rates for *A. penicillioides* and *X. bisporus* at ∼0.656 a_w_ (that is, ≤0.074 and 0.125 mm per day, respectively) were two orders of magnitude slower than those recorded under optimal conditions, yet strains of both species were able to grow down to 0.640 a_w_ (Figures 3 and 4), a hitherto unprecedented limit for mycelial growth of xerophiles. Indeed, at 0.640 a_w_, *A. penicillioides* strain JH06THH and *X. bisporus* strain FRR 3443 grew at rates equivalent to 3.43 and 13.0 mm per year, respectively. In the context of the high-solute and desiccated habitats of xerophiles (some of which can, over periods spanning years or decades, establish populations in environments such as dust and ancient papers and fabrics; Samson and Lustgraaf, 1978; Arai, 2000; Takatori, 2001; Sterflinger and Pinzari, 2012) this is a remarkable rate of growth. Extrapolations indicate lower water-activity limits of 0.636 and 0.632 for *X. bisporus* FRR 3443 and *A. penicillioides* JH06THJ, respectively (Supplementary Table S1 and Figures 3 and 4).

The lowest empirically determined water activity for vegetative growth of a eukaryotic microbe is, therefore, 0.640 and the theoretical lower limit is 0.632 (Figure 4); for Archaea or Bacteria the lowest empirically determined value is 0.635, with theoretically determined minima of down to 0.611 (Figures 1 and 2). Planktonic growth of the yeast *Zygosaccharomyces rouxii* was recorded at 0.620 a_w_ (with a theoretical limit of 0.616; von Schelhorn, 1950), conidial germination of *Aspergillus echinulatus* at 0.620 (Snow, 1949) and germination of *X. bisporus* at 0.605 (Pitt and Christian, 1968), although neither development of mycelium nor sporulation of *X. bisporus* occurred at 0.605 (and none of these achievements have been equalled or exceeded during the subsequent decades, to our knowledge).

### No single domain of life is superior in its tolerance of high-solute substrates

A comparison of 60 of the most solute-tolerant microbes (Figure 5) indicates modest water-activity limits for osmotolerant Bacteria and halotolerant/philic eukaryotes relative to those for halophilic Archaea and Bacteria or xerophilic eukayotes. Empirical determinations of water-activity minima for halophilic prokaryotes indicate that numerous strains are not capable of multiplication below 0.755 a_w_; but that some can do so below 0.650 (Figure 5; Javor, 1984). Up to now, there has been a paucity of studies carried out to establish the true water-activity windows of extreme halophiles by circumventing the solubility limit of NaCl (for example, Table 1 and Figure 1a). Nevertheless, the theoretical determinations suggest parity between the most xerophilic members of the Archaea, Bacteria and Eukarya that are virtually equivalent in their water-activity limits (Figure 5). It is noteworthy that halophilic Archaea and Bacteria are active at water-activity values that are less than both the previously established (Pitt and Christian, 1968; Williams and Hallsworth, 2009) and revised limits for hyphal growth of fungi (0.632, Figure 3) on high-sugar media (Figure 5). If theoretical values (those derived by extrapolation) are not included, the water-activity minimum for multiplication of haloarchaeal strains GN-2 and GN-5 (0.635 a_w_) are marginally lower than those for hyphal growth of xerophilic fungi (Figures 3 and 4). In nature, extremely halophilic Archaea and Bacteria are not only found in salterns but are also present within, and can dominate, microbial communities located in the hypersaline fluid inclusions of salt crystals in evaporite deposits that underlie a considerable portion of the Earth's surface (McGenity *et al.*, 2000; Grant, 2004; Gramain *et al.* 2011). The osmophilic fungal xerophiles *X. bisporus* and *Z. rouxii* (see Figures 4 and 5) inhabit high-sugar environments such as dried fruits (for references, see Lievens *et al.*, 2014). Highly xerophilic strains of *A. penicillioides*, *Aspergillus echinulatus*, *Eurotium amstelodami* and *Eurotium chevalieri* have been isolated from both high-solute and other desiccated habitats (Arai, 2000; Williams and Hallsworth, 2009; Sterflinger and Pinzari, 2012). *Eurotium* species, *Bettsia fastidia*, *A. penicillioides* and *W. sebi* are particularly common in grains, nuts and spices and, indeed, *A. penicillioides* may be the pioneer species in such habitats (Hocking 2003; Pitt and Hocking 2009). These fungi are also common spoilage species in many low water-activity baked foods, dried meats and fish, whereas *Xerochrysium xerophilum*, *Eurotium repens*, *Eurotium halophilicum and W. sebi* species are most commonly associated with high-sugar habitats, particularly confectionery, chocolate, jams, maple syrup, dried substrates such as hay, dry beans and grains, and dried fruits (Pitt and Hocking, 2009; Pitt *et al.*, 2013). Water activity can act as a determinant of community composition and ecosystem function for diverse ecophysiological groups (Grant, 2004; Auguet *et al.*, 2010; Herrera *et al.*, 2012; Bolhuis *et al.*, 2013; Cray *et al.*, 2013a; Zajc *et al.*, 2013). We investigated the limits for algae, fungi and nanoflagellates in saline substrates (Supplementary Table S2) and those for Bacteria in high-sugar or high-polyol substrates (Supplementary Table S3).

The water-activity minima for most halophilic members of the Eukarya ranged between 0.743 and 0.712 (for the fungi *Polypaecilium pisce* at 0.741, *Wallemia ichthyophaga* at 0.720 and *Basipetospora halophila* at 0.712; for the algae *Dunaliella peircei* at 0.743 and *Dunaliella salina* at 0.739; Supplementary Table S2) that is substantially higher than those of comparable species of halophilic Archaea and Bacteria (Figure 5). *Basipetospora halophila* and *Wallemia ichthyophaga* are commonly found in salty environments. Most isolates of *Basipetospora halophila* have been isolated from salted and dried fish and also from dried seaweed food and sea salt (Pitt and Hocking, 2009). *Wallemia ichthyophaga* occurs on salted, dried meat, hypersaline waters of salterns (Zalar *et al.*, 2005), salt crystals and MgCl_2_-rich bitterns (Jančič *et al.*, unpublished data). The hypersaline waters of salterns are also an important habitat for xerophylic *Wallemia sebi* and *Wallemia muriae* (Zalar *et al.*, 2005). *Dunaliella* species are highly prevalent in microbial communities of salt-saturated salterns as well as other niche habitats such as spider-web silk in desert environments (Elevi Bardavid *et al.*, 2008; Azúa-Bustos *et al.*, 2010; Khemakhem *et al.*, 2010; Cray *et al.*, 2013a). The water-activity limits for three nanoflagellates that are also found in salterns at or close to saturated NaCl (0.782 for *Euplaesiobystra hypersalinica*; 0.767 for *Pleurostomum flabellatum*; and 0.757 for *Halocafeteria seosinensis*; see Supplementary Table S2) are lower than those of some other eukaryotes, but are nevertheless exceptional for grazing species that are thought to be absent from most salt-saturated habitats (Elevi Bardavid *et al.*, 2008; Cray *et al.*, 2013a). The xerotolerance of the most sugar-tolerant Bacteria (in the range 0.849 to 0.800 a_w_ for *Mycobacterium parascrofulaceum*, *Mycobacterium smegmatis*, *Saccharibacter floricola* and *Tetragenococcus halophilus*) was inferior to that for the nanoflagellates *E. hypersalinica*, *H. seosinensis* and *P. flabellatum* as well as fungal comparators (Figure 5). *Mycobacterium* species, including those that were capable of growth at high concentrations of glycerol or PEG 400 (down to 0.800; Supplementary Table S3; Santos *et al.*, unpublished data), can be isolated from the surface film of sphagnum moss, algal communities and other habitats that (like salterns) may have high concentrations of glycerol and/or other organic low molecular mass solutes that reduce water activity (Walker *et al.*, 2005; Burkholder *et al.*, 2007; Elevi Bardavid *et al.*, 2008; Kazda and Falkinham, 2009). There are various low water-activity, sugar-rich substances of biotic origin such as dried or high-sugar fruits, honey, maple syrup and sugar-beet juice that can delay or prevent microbial colonisation and the formation of microbial biomass as they depress water activity to values outside the growth windows of most, if not all, osmotolerant and osmophilic Bacteria and yeasts (they may also contain antimicrobials and/or constituents—such as fructose and ethanol—that are chaotropic; for references, see Lievens *et al.*, 2014). It is noteworthy that the data presented for biotic activity at extremely low water-activity (Figure 5) come exclusively from culture-dependent studies as there is a paucity of evidence from culture-independent studies to demonstrate microbial processes at equally low water-activities.

## Concluding remarks

The findings demonstrate that some species of halophilic Archaea and Bacteria are active at water activities considerably below 0.755, suggesting that—based on extant data sets—microbial xerophilicity ultimately converges on a narrow range of water activity (∼0.650–0.600), and possibly even a common value—of ∼0.61—for all three domains of life. Given that saline, rather than sugar-rich, habitats were most common on the early Earth, this finding has implications for the origins of terrestrial life (Stevenson *et al.*, 2014). Furthermore, we know much about the water activity of potential microbial habitats in extraterrestrial locations, some of which could potentially be inhabited by prokaryotic halophiles capable of multiplication below 0.755 a_w_ (Stevenson *et al.*, 2014). There are implications of the findings of the current study, therefore, in relation to planetary protection (Kminek *et al.*, 2010, 2014; Rummel *et al.*, 2014; Stevenson *et al.*, 2014). The net effect of multiple physiological factors and diverse stress parameters is known to determine the extent of microbial growth windows (Hallsworth, 1998; Hallsworth *et al.*, 2003a, 2007; Williams and Hallsworth, 2009; Bhaganna *et al.*, 2010; Chin *et al.*, 2010; Bell *et al.*, 2013; Harrison *et al.*, 2013; Yakimov *et al.*, 2014). Conversely, it appears that water activity is the ultimate determinant for biotic activity and cell division of numerous extremophiles (for example, haloarchaeal strains GN-2 and GN-5, *A. penicillioides* and *X. bisporus*; Figures 1b and 3) and is likely to be equally true for such microbes in their natural habitats, possibly even for microbes not located in high-solute habitats or those that access water from the vapour phase (Rummel *et al.*, 2014; Stevenson *et al.*, 2014). Given the fundamental roles of water as a ‘chaperone' (McCammick *et al.*, 2010) and in macromolecular hydration, generation of hydrophobic forces, and other interactions within and between cellular macromolecules (Franks, 1972; Daniel *et al.*, 2004; Hallsworth *et al.*, 2007; Bhaganna *et al.*, 2010; Ball, 2012; Cray *et al.* 2013b), we consider water to be the most potent force to shape the functional biosphere on Earth (see also Pitt, 1975; Brown, 1976, 1990; Hallsworth *et al.*, 2003b; Grant, 2004; Hallsworth *et al.*, 2007; Williams and Hallsworth, 2009; Cray *et al.*, 2013a; Yakimov, *et al.*, 2014). Converging lines of evidence indicate that the ultimate limits for solute tolerance of xerophilic fungi and halophilic Archaea and Bacteria are determined by a prohibitively high energy expenditure that is required for stress adaptation (Hocking, 1993; Oren, 1999; Park *et al.*, 2006; Arino *et al.*, 2010; Cray *et al.*, 2013a). It is therefore noteworthy that *Aspergillus* species, which appear to have an extraordinary capacity for energy generation (Flipphi *et al.*, 2009; Cray *et al.*, 2013a), also have exceptional tolerances to diverse stresses and an ability to out-compete other microbes, thereby dominating their respective habitats (Cray *et al.*, 2013a). In other words, common physicochemical and/or thermodynamic constraints determine this limit, irrespective of phylogeny.

It may be that a greater understanding of microbial stress biology in relation to low water-activity habitats can lead to further improvements in food preservation, biological control (Cray *et al.*, 2015), management of soil microbiology in arid regions and interventions to enhance crop plant/mycorrhizae (and other plant/microbe) interactions or plant–insect interactions mediated by osmophilic microbes inhabiting plant nectar (Raguso, 2004; Vannette *et al.*, 2012; Herrera *et al.*, 2013; Good *et al.*, 2014); thereby enhancing plant conservation, crop production and, ultimately, global food security. The majority of studies carried out to determine limits of microbial solute tolerance have focused on culturable species of weed-like and/or copiotrophic microbes (Figures 1, 2, 3; Cray *et al.*, 2013a; Oren and Hallsworth, 2014). As a consequence, little is known about water-activity limits for oligotrophic and/or slow-growing species that may undergo a single cell division over a period of decades or longer (Parkes *et al.*, 2000; Lomstein *et al.*, 2012; Rummel *et al.*, 2014). Other questions remain outstanding, such as: what are the metabolic factors that ultimately limit energy generation as well as the synthesis and retention of compatible solutes; can *in vitro* studies of transcription (Youseff *et al.*, 2014) or culture-independent techniques to detect metabolic activity (Hallsworth *et al.*, 2007; Mosier *et al.*, 2013; Yakimov *et al.*, 2014) offer insights into the *in-situ* water-activity limits for microbial communities in hostile environments; why are halophilic eukaryotes and osmotolerant/philic prokaryotes relatively intolerant to high solute-concentrations; what are the phenotypic differences between strains of a single bacterial species isolated from salt-rich environments, that are unable to grow at high-sugar concentrations, and those isolated from sugar-rich substrates that can do so (Justé *et al.*, 2008a, 2008b); how do the limitations of certainty and accuracy of water-activity quantification (Stevenson *et al.*, 2014) compare with those of chaotropicity (Cray *et al.*, 2013b), pH and temperature determination in relation to the sensitivity of the cellular system; can further manipulations of environmental chemistry (e.g. Harrison *et al.*, 2014) lead to microbial life at solute concentrations currently thought to be prohibitive; and could synthetic biology be utilised to obtain microbial cell(s) capable of completing life cycles under hitherto nonpermissive conditions? We are intrigued to see whether further studies to address some of these questions can potentially lead to the documentation of life processes at <0.600 a_w_.

## Figures and Tables

**Table 1:** Doubling times for halophilic Archaea and Bacteria in media supplemented with NaCl (or NaCl plus other solutes) to give a range of water-activity values (see Supplementary Table S4)^a^

*Species of halophile (strain designation*^b^)	*Source of isolate*	*Water activity of medium*^c^								
		*0.803*	*0.775*	*0.717*	*0.712*	*0.709*	*0.693*	*0.687*	*0.681*	*0.642*
*Bacteria*										
*Pontibacillus* (strain AS2)^d^	Deep-sea sediment^e^	ND	<7 Days	NG	NG	ND	NG	NG	NG	NG
*Salinicola* (strain LC26)^f^	Deep sea^g^	ND	<7 Days	NG	NG	ND	NG	NG	NG	NG

*Archaea*										
*Halobacterium noricense* (DSM 15987)	Permian rock salt^h^	<7 Days	ND	∼14 Days	NG	ND	NG	∼14 Days	NG	NG
*Halobacterium* sp. NRC-1	Not documented	<7 Days	ND	∼21 Days	NG	ND	NG	NG	NG	NG
*Halococcus morrhuae* (NCIMB 787)	The Dead Sea^i^	<7 Days	ND	∼21 Days	NG	ND	NG	NG	NG	NG
*Halococcus salifodinae* (DSM 13046)	Halite deposit^j^	<7 Days	ND	∼21 Days	NG	ND	∼21 Days	NG	NG	NG
*Haloquadratum walsbyi* (DSM 16790)	Saltern^k^	ND	ND	ND	ND	<7 Days^l^	ND	ND	ND	ND
*Halorubrum sacchar ovorum* (NCIMB 2081)	Saltern^m^	<7 Days	ND	NG	NG	ND	NG	NG	NG	NG
*Natrinema pallidum* (NCIMB 777)	Salted cod	<7 Days	ND	NG	NG	ND	NG	NG	∼14 Days	NG

Abbreviations: ND, not determined; NG, no measurable growth occurred.

a Liquid nutrient media were supplemented with NaCl, NaCl plus MgCl_2_ or NaCl plus MgCl_2_ plus glycerol or ethylene glycol; for details of medium compositions see Supplementary Table S4. Incubations were carried out at 20 °C for *Pontibacillus* strain AS2 and *Salinicola* strain LC26 and at 37 °C for all other species. Assessments of cell density were carried out over a period of three months (see Materials and methods).

b For the source of cultures see *Supplementary Materials and Methods*.

c Water-activity values were determined for each medium at the relevant incubation temperature and as described in the Materials and methods. For details of medium composition see Supplementary Table S4.

d Formerly known as *Bacillus* strain AS2 (see Sass *et al.*, 2008).

e Sediment (upper 2 cm) beneath a NaCl-dominated, hypersaline brine lake in the L'Atalante Basin, Mediterranean Sea (Sass *et al.*, 2008).

f Formerly known as *Halomonas* strain LC26 (see Daffonchio *et al.*, 2006).

g Brine lake/seawater interface of NaCl-dominated, hypersaline brine lake in the Bannock Basin, Mediterranean Sea (Sass *et al.*, 2008).

h Halite obtained from 470-m-deep bore cores into a salt mine at Altaussee, Austria (Gruber *et al.*, 2004).

i Israel (Tindall, 1992); *Halococcus morrhuae* was formerly known as *Sarcina morrhuae* (Kocur and Hodgkiss, 1973).

j Obtained via solution-mining of Triassic halite (Lostock, UK) in a saturated NaCl brine (Norton *et al.*, 1993).

k Salt-saturated crystalliser pond of a solar saltern in Braç del Port, Alicante, Spain (Bolhuis *et al.*, 2004).

l Growth data obtained from Bolhuis *et al.* (2004); medium water-activity determined as described in Materials and methods.

m Mixture of mud and brine in saltern, southern section of San Francisco Bay, California, USA (Tomlinson and Hochstein, 1976).
