# Supplementary material for: Is there a common water-activity limit for the three domains of life?
Source: ISME J. 2014 Dec 12;9(6):1333–51. doi: 10.1038/ismej.2014.219 (PMC4438321; doi:10.1038/ismej.2014.219)
Supplement: Supplementary Information [file ismej2014219x1.doc]

**Supporting Information**

**Contents**

- Supplementary materials and methods
- Table S1
- Table S2
- Table S3
- Table S4
- Table S5
- Table S6
- Table S7
- Table S8
- Supporting references

**Supplementary materials and methods**

*Organisms and media used in previous studies*

For the source of ***Pontibacillus* strain AS2 (formerly** *Bacillus* strain AS2) see **Sass *et al*. (2008) and for** *Salinicola* **strain LC26 (formerly *Halomonas* strain LC26) see Daffonchio *et al*. (2006). The source and composition of media for *Haloquadratum walsbyi* (DSM 16790) are detailed in Bolhuis *et al*. (2004); see also Table S4.** Cultures were maintained in Payne’s medium (Payne *et al*., 1960) with the exception of *Hqr. walsbyi* (see Bolhuis *et al*., 2004) and incubated at a temperature of 37°C for all species. **The compositions for the salt-supplemented culture media used to assay these halophiles are detailed in Table S4. Cultures were incubated** at 20°C for ***Pontibacillus* strain AS2** and *Salinicola* **strain LC26, and at** 37°C for ***Hqr. walsbyi***. For studies of NaCl tolerance, *S. ruber* was maintained in a complex medium supplemented with NaCl (see Table S6; Peña *et al*. 2010; Sher *et al*. 2004). **Sources of other strains and** composition of culture media **are detailed in the following articles: for** haloarchaeal strains GN-2 and GN-5, cultured in bittern brines supplemented with peptone at 37°C for 6 d (calculated and re-plotted against water activity; Fig. 1b) **in Javor (1984); for the** mixed community of halophilic Bacteria from a crystallizer pond, cultured in a complex medium with supplemented NaCl incubated at 37°C (calculated and re-plotted against water activity; Fig. 1c) in Antón *et al*. (2000)**; for the Bacteria** *Halorhodospira halochloris* (strain not specified), and *Halorhodospira halophila* strain (DSM 244^T^) cultured in a defined medium supplemented with NaCl (calculated and re-plotted against water activity; Fig. 2a and b) in Deole *et al*. (2013), *Halanaerobium lacusrosei* (strain DSM 10165^T^) cultured in a complex medium supplemented with NaCl and incubated at 37°C (calculated and re-plotted against water activity), in Cayol *et al*. (1995), and *Actinopolyspora halophila* (strain ATCC 27976^T^) cultured in a complex medium supplemented with NaCl incubated at 37°C for 14 days (calculated and re-plotted against water activity), in Yoshida *et al*. (1991); the Archaea ‘Haloarcula californiae’ (strain DSM 8905) and ‘Haloarcula sinaiiensis’ (strain DSM 8928) cultured in a complex medium supplemented with NaCl and incubated at 37°C (calculated and re-plotted against water activity), in Javor *et al*. (1982), and *Halorhabdus utahensis* (strain DSM 12940^T^) cultured in a defined medium supplemented with NaCl and incubated at 30°C (calculated and re-plotted against water activity), in Wainø *et al*. (2000).

*Utilization of growth-related data from previous studies*

For *Hqr. walsbyi*, cell division at high salt concentration was determined previously (Table S1; Bolhuis *et al*., 2004) and the culture medium (see Table S4) was recreated for water-activity determination which as described below. Growth rates of haloarchaeal strains GN-2 and GN-5 within sterilised bittern brines of supplemented with peptone were previously determined via spectrophotometry (OD_750nm_); Javor *et al*. (1984). The density values (°Bé) of these brines were converted to specific gravity via the equation: specific gravity = 145/145-°Bé. The specific-gravity values were then converted to water activity using a standard specific gravity versus water activity curve (Javor, 1989), in order to plot biomass (OD_750nm_) against water activity (Fig. 1b). Development of bacterial communities in complex media supplemented with a range of salt concentrations (Fig. 1c) was assessed by FISH (using halophilic Bacteria specific probe EHB412) and DAPI staining (Antón *et al*. 2000). Data for cells (medium ml)^-1^ at various time intervals (Antón *et al*. 2000) were used to determine doubling d^-1^ in relation to the exponential growth phase for both halophilic Bacteria and total bacterial populations at each salt concentration (Fig. 1c). The media for each salt concentration were recreated to carry out water-activity determinations (see below; Fig. 1c). NaCl tolerance of *S. ruber* was plotted using extant datasets (Peña *et al*. 2010; Sher *et al*. 2004), from which growth rates were calculated during exponential-phase to give an average increase in OD units d^-1^. Media used in these studies were recreated and measured for their water activities at 35**°C (see below; Table S6).**

**Growth of the Bacteria** *Hlr. halochloris* and *Hlr. halophila* was assessed by quantifying OD_600nm_ in a defined medium supplemented with a range of NaCl concentrations at various time-intervals (Deole *et al*. 2013). Water activity was calculated according to the concentrations of NaCl and other medium components (see below) in order to plot cell doubling d^-1^ against water activity (Figs. 2a and 2b). **Growth of the bacterium** *H. lacusrosei* (37°C) was assessed by quantifying OD_660nm_ in complex media supplemented with NaCl (Cayol *et al*., 1995). Water activity was calculated according to the concentrations of NaCl and other medium components (see below) in order to plot OD units d^-1^ against water activity (Figs. 2c). For the bacterium *A. halophila*, growth was assessed (after 14 days at 37°C) in a complex liquid medium supplemented with NaCl (Yoshida *et al*. 1991) after which the total mycelial dry weight was determined (mg ml^-1^). Water activity was calculated according to the concentrations of NaCl and other medium components (see below) in order to plot OD units d^-1^ against water activity (Fig 2d). Growth rates of the Archaea ‘Har. californiae’ and ‘Har. sinaiiensis’ (37°C) were determined by quantifying OD_750nm_ in complex media supplemented with NaCl (Javor *et al*., 1982). Water activity was calculated according to the concentrations of NaCl and other medium components (see below) in order to plot growth rates, as fraction of optimal rate (µ'), against water activity (Fig. 2e). **Growth of the archaeon** *Hrd. utahensis* (30°C) was assessed by quantifying OD_600nm_ in a defined medium supplemented with NaCl (Wainø *et al*., 2000). Water activity was calculated according to the concentrations of NaCl and other medium components (see below) in order to plot growth rates (OD units d^-1^) against water activity (Fig. 2f).

**Table S1.** Source of data which indicate ability to grow at high solute concentrations, and regression analyses for extrapolation of water-activity curves (see *Materials and methods*).

| **Microbial species (strain designation) or community** | **Location of data within current article** | **Original source of microbial growth data** | **Original source of water-activity values** | **Regression analysis (r^2^ value)** |
| --- | --- | --- | --- | --- |
| *Actinopolyspora halophila* (ATCC 27976^T^) | Figs. 2d and 5 | Yoshida *et al*. (1991) | NaCl concentrations (Yoshida *et al*., 1991) were converted to water-activity values using a standard curve (see *Materials and methods*) | Modified Gaussian, 4 parameter (0.9985) |
| *Asaia bogorensis* **(JCM 10569**^T^**)** | Tables S3; Fig. 5 | **Jojima *et al.* (2004)** | Glucose concentrations (**Jojima *et al.* 2004)** were converted to water-activity values using a standard curve (see *Materials and methods*) | Polynomial, 5^th^ order (1) |
| ***Aspergillus*** *echinulatus*^a^ | Fig. 5 | Snow (1949) | Values of relative humidity (Snow, 1949) were converted mathematically to water activity^b^ | *not applicable* |
| ***Aspergillus penicilliodes* (FRR 2612)** | **Table S2;** Fig. 5 | Pitt and Hocking (1977) | Pitt and Hocking (1977) | *not applicable* |
| *Aspergillus penicillioides*^a^ | Fig. 5 | Pitt and Hocking (2009) | Pitt and Hocking (2009) | *not applicable* |
| *Aspergillus penicillioides* (JH06THH)  *Aspergillus penicillioides*  (JH06THJ)  ***Bacillus cereus* (5B)**  ***Bacillus megaterium* (KM)**  ***Bacillus sphaericus***^a^  ***Bacillus subtilis* (W168; ATCC 6051)** | Table S8; Figs. 3 and 5  Table S8; Figs. 3 and 5  Table S2  Table S2  Table S2  Table S2 | *Current study*  *Current study*  **Marshall *et al*.**  **(1971)**  **Marshall *et al*. (1971)**  **Marshall *et al*. (1971)**  **Marshall *et al*. (1971)** | Water-activity values of media were determined empirically^b^  Water-activity values of media were determined empirically^b^  **Marshall *et al*. (1971)**  **Marshall *et al*. (1971)**  **Marshall *et al*. (1971)**  **Marshall *et al*. (1971)** | Polynomial, 2^nd^ order (0.9898)  Polynomial, 5th order (0.9898)  *not applicable*  *not applicable*  *not applicable*  *not applicable* |
| ***Basipetospora halophila* (FRR 2787)** | Table S2; Fig. 5 | **Wheeler *et al*. (1988)** | NaCl concentrations (Wheeler *et al*., 1991) were converted to water-activity values using a standard curve (see *Materials and methods*) | Polynomial, 5^th^ order (0.9951) |
| *Bettsia fastidia*^a^ | Fig. 5 | Pitt and Christian (1968) | Pitt and Christian (1968) | *not applicable* |
| *Cladosporium sphaerospermum* (EXF-2037)  ***Clostridium botulinum* (type-A strain)** | Table S2  Table S2 | Zalar *et al*. (2007)  **Marshall *et al*. (1971)** | NaCl concentrations (Zalar *et al*., 2007) were converted to water-activity values using a standard curve (see *Materials and methods*)  **Marshall *et al*. (1971)** | *not applicable*  *not applicable* |
| *Debaryomyces hansenii* (**DSM 70590)** | Table S2; Fig. 5 | *Current study* | Water-activity values of media were determined empirically^b^ | *not applicable* |
| *Dunaliella parva* (UTEX 1983),  *Dunaliella peircei* (UTEX 2192)  *Dunaliella salina* (UTEX 200) | Table S2; Fig. 5  Table S2; Fig. 5  Table S2; Fig. 5 | Cifuentes *et al*. (2001)  Cifuentes *et al*. (2001)  Cifuentes *et al*. (2001) | NaCl concentrations (Cifuentes *et al*., 2001) were converted to water-activity values using a standard curve (see *Materials and methods*)  NaCl concentrations (Cifuentes *et al*., 2001) were converted to water-activity values using a standard curve (see *Materials and methods*)  NaCl concentrations (Cifuentes *et al*., 2001) were converted to water-activity values using a standard curve (see *Materials and methods*) | Polynomial, 4^th^ order (0.9875)  Polynomial, 3^rd^ order (0.9999)  Linear (0.9689)^c^ |
| *Euplaesiobystra hypersalinica* (CCAP 1528/1) | Table S2; Fig. 5 | Park *et al*. (2009) | Media were recreated (Park *et al*., 2009) and water activities were determined empirically (see *Materials and methods*) | *not applicable* |
| *Eurotium amstelodami* (FRR 2792 and FRR 0475) | Fig. 5 | Williams and Hallsworth (2009) | Williams and Hallsworth (2009) | *not applicable* |
| *Eurotium chevalieri* (JH06THI) | Fig. 5 | Williams and Hallsworth (2009) | Williams and Hallsworth (2009) | *not applicable* |
| *Eurotium halotolerans* (MZKI A-560 and EXF-4356) | Table S2; Fig. 5 | Butinar *et al*. (2005) | NaCl concentrations (Butinar *et al*., 2005) were converted to water-activity values using a standard curve (see *Materials and methods*) | *not applicable* |
| *Eurotium halophilicum* (FRR 2471) | Fig. 5 | Andrews and Pitt (1987) | Andrews and Pitt (1987) | *not applicable* |
| *Eurotium repens* (JH06JPD) | Fig. 5 | Williams and Hallsworth (2009) | Williams and Hallsworth (2009) | *not applicable* |
| *Eurotium rubrum* (FRR 0326) | Fig. 5 | Gock *et al*. (2003) | Gock *et al*. (2003) | *not applicable* |
| ***Gluconacetobacter diazotrophicus* (DSM 5601**^T^**)** | Table S3; Fig. 5 | **Jojima *et al.* (2004)** | Glucose concentrations (**Jojima *et al.* 2004)** were converted to water-activity values using a standard curve (see *Materials and methods*) | *not applicable* |
| *Halanaerobium lacusrosei* (DSM 10165^T^) | Figs. 2c and 5 | Cayol *et al*. (1995) | NaCl concentrations (Cayol *et al*., 1995) were converted to water-activity values using a standard curve (see *Materials and methods*) | Polynomial, 4^th^ order (0.9819) |
| Haloarchaeal strain GN-2  Haloarchaeal strain GN-5 | Figs. 1b and 5  Figs. 1b and 5 | Javor *et al*. (1984)  Javor *et al*. (1984) | The density values (°Bé) of brines (Javor *et al*., 1984) were converted to specific gravity via the equation: 145/145-°Bé = specific gravity. In turn, specific-gravity was converted to water activity using a standard curve (Javor, 1989)  The density values (°Bé) of brines (Javor *et al*., 1984) were converted to specific gravity via the equation: 145/145-°Bé = specific gravity. In turn, specific-gravity was converted to water activity using a standard curve (Javor, 1989) | Polynomial, 6^th^ order (0.9971)  Linear^c^ (0.9166) |
| ‘Haloarcula californiae’  (DSM 8905)  ‘Haloarcula sinaiiensis’  (DSM 8928) | Figs. 2e and 5  Figs. 2e and 5 | Javor *et al*. (1982)  Javor *et al*. (1982) | NaCl concentrations (Javor *et al*., 1982) were converted to water-activity values using a standard curve (see *Materials and methods*)  NaCl concentrations (Javor *et al*., 1982) were converted to water-activity values using a standard curve (see *Materials and methods*) | Linear ^c^ (0.9912)  Linear^c^ (0.9772) |
| *Halobacterium noricense* (**DSM 15987^T^**)  *Halobacterium* sp*.* NRC-1 | Table 1 and S4; Figs. 1a and 5  Table 1 and S4; Figs. 1a and 5 | *Current study*  *Current study* | Water-activity values of media were determined empirically^b^  Water-activity values of media were determined empirically^b^ | *not applicable*  *not applicable* |
| *Halobacterium* strain (004.1) | Table S5; Figs. 1d and 5 | *Current study* | Water-activity values of media were determined empirically^b^ | Polynomial, 3^rd^ order (0.9829) |
| *Halocafeteria seosinensis* (EHF34) | Table S2 | Park *et al*. (2006) | Media were recreated (Park *et al*., 2009) and water activities were determined empirically (see *Materials and methods*) | *not applicable* |
| *Halococcus morrhuae* **(NCIMB**  **787^T^)**  *Halococcus salifodinae*  **(DSM 13046)** | Table 1 and S4; Figs. 1a and 5  Table 1 and S4; Figs. 1a and 5 | *Current study*  *Current study* | Water-activity values of media were determined empirically^b^  Water-activity values of media were determined empirically^b^ | *not applicable*  *not applicable* |
| *Halorhabdus utahensis* (DSM 12940^T^) | Figs. 2f and 5 | Wainø *et al*. (2000) | NaCl concentrations (Wainø *et al*., 2000) were converted to water-activity values using a standard curve (see *Materials and methods*) | Modified Gaussian, 5 parameter (0.9723) |
| *Halorhodospira halochloris*^a^  *Halorhodospira halophila*  (DSM 244^T^) | Figs. 2a and 5  Figs. 2a and 5 | Deole *et al*. (2013)  Deole *et al*. (2013) | Media were recreated (Deole *et al*., 2013) and water activities were determined empirically (see *Materials and methods*)  Media were recreated (Deole *et al*., 2013) and water activities were determined empirically (see *Materials and methods*) | Linear^c^ (0.9679)  Polynomial, 2^nd^ order (0.9713) |
| *Halorubrum saccharovorum* **(NCIMB 2081^T^)** | Table 1 and S4; Fig. 1a | *Current study* | Water-activity values of media were determined empirically^b^ | *not applicable* |
| Halophilic bacterial community (largely *Salinibacter* spp.) | Table S6; Figs. 1c and 5 | Antón *et al*. (2000) | Media were recreated (Antón *et al*., 2000) and water activities were determined empirically (see *Materials and methods*) | Polynomial, 3^rd^ order (1); linear (0.9088)^d^ |
| *Haloquadratum walsbyi* **(DSM 16790)** | Table 1 and S4; Figs. 1a and 5 | Bolhuis *et al*. (2004) | Media were recreated (Bolhuis *et al*., 2004) and water activities were determined empirically (see *Materials and methods*) | *not applicable* |
| ***Halotalea alkalilenta* (AW-7**^T^**)** | Table S3; Fig. 5 | **Ntougias *et al*. (2007)** | Glucose concentrations **(Ntougias *et al*., 2007)** were converted to water-activity values using a standard curve (see *Materials and methods*) | *not applicable* |
| ***Hortaea werneckii* (**EXF-225)  *Lactobacillus plantarum*^a^ | Table S2; Fig. 5  Table S3 | Gunde-Cimerman *et al*. (2000)  **Troller and Stinson (1981)** | NaCl concentrations (Wainø *et al*., 2000) were converted to water-activity values using a standard curve (see *Materials and methods*)  Glycerol concentrations were converted to water-activity values using a standard curve (see *Materials and methods*) | *not applicable*  Polynomial, 2^nd^ order (0.9801) |
| ***Lactococcus lactis*^a^** | Table S3; Fig. 5 | **Troller and Stinson (1981)** | Glucose concentrations were converted to water-activity values using a standard curve (see *Materials and methods*) | Polynomial, 4^th^ order (0.9875) |
| ***Micromonospora* sp.**  **(JCM 3050)**  ***Micromonospora grisea***  **(JCM 3182),**  ***Mycobacterium parascrofulac****eum* (**LAIST_NPS017)**  ***Mycobacterium smegmatis* (ATCC 10143)** | Table S3; Fig. 5  Table S3; Fig. 5  Table S3; Fig. 5  Table S3; Fig. 5 | Stevenson *et al*. (2014)  Stevenson *et al*. (2014)  Santos *et al*. (unpublished data)  Santos *et al*. (unpublished data) | Stevenson *et al*. (2014)  Stevenson *et al*. (2014)  Santos *et al*. (unpublished data)  Santos *et al*. (unpublished data) | *not applicable*  *not applicable*  *not applicable*  *not applicable* |
| ***Natrinema pallidum* (NCIMB 777^T^)** | Table 1 and S4; Figs. 1a and 5 | *Current study* | Water-activity values of media were determined empirically^b^ | *not applicable* |
| ***Pleurostomum flabellatum* (CCAP 1959/1)** | Table S2; Fig. 5 | Park *et al*. (2007) | Media were recreated (Park *et al*., 2007) and water activities were determined empirically (see *Materials and methods*) | Linear (1)^c^ |
| ***Polypaecilum pisce* (FRR 2733)** | Table S2; Fig. 5 | **Wheeler *et al*. (1988)** | NaCl concentrations (Wheeler *et al*., 1998) were converted to water-activity values using a standard curve (see *Materials and methods*) | Polynomial, 2^nd^ order (0.9617) |
| ***Pontibacillus* (AS2)**  *Rosenbergiella nectarea* (8N4; LMG 26121; DSM 24150) | Table 1 and S4; Figs. 1a and 5  Table S2 | Bolhuis *et al*. (2004)  Halpern *et al*., (2013) | Media were recreated (Bolhuis *et al*., 2007) and water activities were determined empirically (see *Materials and methods*)  Sucrose concentrations (**Halpern *et al.* 2013)** were converted to water-activity values using a standard curve (see *Materials and methods*) | *not applicable*  *not applicable* |
| ***Saccharibacter floricola* (**DSM 15669^T^**)** | Table S3; Fig. 5 | **Jojima *et al.* (2004)** | Glucose concentrations (**Jojima *et al.* 2004)** were converted to water-activity values using a standard curve (see *Materials and methods*) | Linear (0.9874)^c^ |
| *Salinibacter ruber* (DSM 13855**^T^**) | Table S6; Figs. 1e and 5 | Sher *et al*. (2004); Peña *et al*. (2010) | Media were recreated (Sher *et al*., 2004; Peña *et al*. 2010) and water activities were determined empirically (see *Materials and methods*) | Linear (1)^c^ ; polynomial, 4^th^ order (1) |
| *Salinicola* (LC26) | Table 1 and S4; Figs. 1a and 5 | *Current study* | Media were recreated (Bolhuis *et al*., 2004) and water activities were determined empirically (see *Materials and methods*) | *not applicable* |
| *Salisaeta longa* (DSM 21114**^T^**) | Fig. 1f | *Current study* | Water-activity values of media were determined empirically^b^ | Polynomial, 2^nd^ order (1)^c^ ; polynomial, 3^rd^ order (1)^d^ |
| ***Sarcina* sp. (2b)** | Table S3; Fig. 5 | **Marshall *et al*. (1971)** | **Marshall *et al*. (1971)** | *not applicable* |
| ***Staphylococcus aureus***  **(ATCC 6538P, NA and FM1)** | Table S3; Fig. 5 | **Scott (1953)** | Sucrose concentrations **(Scott, 1953)** were converted to water-activity values using a standard curve (see *Materials and methods*) | *not applicable* |
| ***Staphylococcus epidermidis*^a^** | Table S3; Fig. 5 | **Marshall *et al*. (1971)** | **Marshall *et al*. (1971)** | *not applicable* |
| ***Streptomyces albidoflavus***  **(JCM 4198^T^)**  ***Streptomyces rectiviolaceus* (JCM 9092^T^)** | Table S3; Fig. 5  Table S3; Fig. 5 | Stevenson *et al*. (2014)  Stevenson *et al*. (2014) | Stevenson *et al*. (2014)  Stevenson *et al*. (2014) | *not applicable*  *not applicable* |
| *Tetragenococcus halophilus*  (T11 and T15) | Table S3; Fig. 5 | Justé *et al*. (2008a; 2008b) | Sucrose solutions were created based on °Bx values (Justé *et al*. 2008a; 2008b) and water activities were determined empirically | *not applicable* |
| *Wallemia ichthyophaga*  (EXF-994) | Table S2; Fig. 5 | Zalar *et al*. (2005) | NaCl concentrations (Zalar *et al*., 2005) were converted to water-activity values using a standard curve (see *Materials and methods*) | Polynomial, 3^rd^ order (0.9963)^c^ |
| *Wallemia sebi* (FRR 1473) | Table S2; Fig. 5 | Pitt and Hocking (1977); Zalar *et al*. (2005) | Pitt and Hocking (1977); NaCl concentrations (Zalar *et al*., 2005) were converted to water-activity values using a standard curve (see *Materials and methods*) | *not applicable* |
| *Wallemia muriae* (EXF-951) | Table S2; Fig. 5 | Zalar *et al*. (2005) | Water activity was calculated according to the concentrations of NaCl and other medium components (see *Materials and methods*) | Polynomial, 4^th^ order (0.9810) |
| *Xeromyces bisporus* (FRR 0025) | Fig. 5 | **Pitt and Christian (1968)** | Pitt and Christian (1968) | *not applicable* |
| *Xeromyces bisporus* (FRR 0025, FRR 2347 and FRR 3443) | Table S8; Figs. 4 and 5 | Current study | Water activities of media were determined empirically (see *Materials and methods*) | *not applicable* |
| *Xerochrysium xerophilum*  (CBS 153.67^T^) | Fig. 5 | Leong *et al*. (2011) | Leong *et al*. (2011) | Polynomial, 4^th^ order (1) |
| ***Zygosaccharomyces rouxii*^a^**  ***Zymomonas mobilis* (22 strains)** | Fig. 5  Table S2 | **von Schelhorn (1950)**  **Swings and De Ley (1977)** | **von Schelhorn (1950)**  Glucose concentrations **(Scott, 1953)** were converted to water-activity values using a standard curve (see *Materials and methods*) | Linear^c^ (0.9999)  *not applicable* |

a. Strain designation not specified.

b. Water-activity values are equivalent to equilibrium-relative humidity values multiplied by a factor of 0.01 (Brown, 1990).

c. Linear regression analyses were carried out using data-points at water-activity values which corresponded to optimum and sub-optimum growth only.

d. Anomalous values were excluded from the analysis.

**Table S2.** Water-activity (a_w_) limits for the biotic activity^a^ of extremely xerophilic microbial eukaryotes on saline substrates^b^.

**Microbial species Substrate or cult- Lowest a_w_ at Lower a_w_ Notes and references**

**(strain[s]) ure medium, and which biotic limit for**

**added stressor(s) activity was biotic activity**

**empirically derived by**

**determined^c^  extrapolation^d^**

**_____________________________________________________________________________________________________________________________________________________________________**

**Green micro-algae**

***Dunaliella parva*  Johnson’s (J/1) 0.774 (25**°**C) 0.758 Growth rates were quantified over a range of NaCl concns. up to**

**(UTEX 1983) Medium supplem-**  **30% w/v (0.774 a_w_). Extrapolation of the curve** indicates a **theo-**

ented with glycerol retical growth limit of 31.8% w/v (0.758 **a_w_; Cifuentes *et al*.,**

**2001)**

***Dunaliella peircei* Johnson’s (J/1) 0.774 (25**°**C) 0.743 Growth rates were quantified over a range of NaCl concns. up to**

**(UTEX 2192) Medium supplem-**  **30% w/v (0.774 a_w_). Extrapolation of the curve** indicates a **theo-**

ented with glycerol retical growth limit of 33.3% w/v (0.743 **a_w_; Cifuentes *et al*.,**

**2001)**

***Dunaliella salina* Johnson’s (J/1) 0.774 (25**°**C) 0.739 Growth rates were quantified over a range of NaCl concns. up to**

**(UTEX 2192) Medium supplem-**  **30% w/v (0.774 a_w_). Extrapolation of the curve indicates a theo-**

ented with glycerol retical growth limit of 33.7% w/v (0.739 **a_w_; Cifuentes *et al*.,**

**2001)**

**Ascomycete yeasts and fungi**

***Aspergillus penicilliodes* Yeast Nitrogen Glu- 0.747 (25°C) na The minimum water activity for germination on high-NaCl media
 (FRR 2612) cose Agar supple- was 0.747; germination occurred after 8.3 days (Pitt and Hock-**

**mented with NaCl ing, 1977)**

***Basipetospora halophila* Malt Extract, Yeast 0.746 (30°C) 0.712 Growth rates were quantified over a range of NaCl concns. up to**

**(FRR 2787) Extract Phosphate saturation (0.746 a_w_). Extrapolation of the curve suggests gro-**

**Agar supplemented wth down to 0.700 a_w_, while studies of conidial germination ind-**

with NaCl **icate a** limit of ≤ **0.700 a_w_ (Wheeler *et al*., 1988)**

*Cladosporium* Malt Extract Agar **0.864** (25°**C**) na Growth was observed after 7 d on medium containing 3.42 M

*sphaerospermum* (EXF- supplemented with NaCl (Zalar *et al*., 2007)

2037) NaCl

***Debaryomyces hansenii* Yeast Extract, Cas- 0.803 (**37°**C) <0.803 Growth observed after 16 days on media containing 3.5 M NaCl**

**(DSM 70590) amino Acid, Tri So- and 0.4 M MgCl_2_ (0.803 a_w_). See** *Materials and methods*

**dium Citrate Broth**

**supplemented with**

**NaCl and MgCl_2_**

*Hortaea werneckii*  Malt Extract Agar **0.780** (25°**C**) na *Hortaea werneckii* was isolated from a saltern onto agar

(EXF-225) supplemented with containing 5.48 M NaCl (Gunde-Cimerman *et al*., 2000)

NaCl

***Eurotium halotolerans* Malt Extract Agar 0.752 (**25°**C) na Growth rates were quantified over a range of NaCl concns. up to**

(EXF-4356) supplemented with  **32.5% w/v (0.751 a_w_; Butinar *et al*., 2005)**

NaCl

***Polypaecilum pisce* Malt Extract, Yeast 0.746 (**30°**C) 0.741 Growth rates were quantified over a range of NaCl concns. up to**

**(FRR 2733) Extract Phosphate saturation (0.746 a_w_). Extrapolation of the curve suggests**

**Agar** supplemented growth **down to 0.700 a_w_, while studies of conidial germination**

with NaCl **indicate a** limit of **0.746 a_w_ (Wheeler *et al*., 1988)**

Basidiomycete fungi

***Wallemia ichthyophaga* Malt Extract, Yeast 0.771 (**24°**C) 0.720 Growth rates were quantified over a range of NaCl concns. (down**

**(EXF-994) Extract Phosphate to 0.771 a_w_). Extrapolation of the curve suggests growth down to**

**Agar** supplemented **down to 0.697 a_w_ (Zalar *et al*., 2005)**

with NaCl

***Wallemia sebi* Yeast Nitrogen Glu-0.751 (**25°**C) na The minimum water activities for germination and growth on high-**

**(FRR 1473) cose Agar supple- NaCl media were found to be 0.751 and 0.805 respectively (Pitt**

mented with NaCl **and Hocking, 1977;** Zalar *et al*., 2005). Extrapolation of the gro-

wth curve suggests growth down to 0.785 a_w_.

***Wallemia muriae* Malt Extract, Yeast 0.805 (**24°**C) 0.795**  The minimum water activity at which growth on high-NaCl media

**(EXF-951) Extract Phosphate has been observed is 0.805 (Zalar *et al*., 2005). Extrapolation of Agar** supplemented **the curve suggests growth down to 0.795 a_w_**

with NaCl

**Heterotrophic nanoflagellates**

*Euplaesiobystra hypers-* **Artificial Seawater** 0.782 (37°**C) na Growth rates were quantified on high-salt, NaCl dominated media**

*alinica* **(**CCAP 1528/1) **Broth supplemented up to 300‰ salinity (0.782 a_w_). The organism was isolated from**

**with NaCl and other a solar saltern with** 300**‰ salinity (Park *et al*., 2009)**

**salts^e^**

***Halocafeteria seosinen-* Artificial Seawater 0.757** (35°**C) na Growth rates were quantified on high-salt, NaCl dominated media**

***sis* (**EHF34) **Broth supplemented up to 363‰ salinity (0.757 a_w_). The organism was isolated from**

**with NaCl and other a solar saltern with** 300**‰ salinity (Park *et al*., 2006)**

**salts^e, f^**

***Pleurostomum flabella-* Artificial Seawater 0.795 (40**°**C) 0.767 Growth rates were quantified on high-salt, NaCl dominated media**

***tum* (**CCAP 1959/1) **Broth supplemented up to 313‰ salinity (0.795 a_w_). The organism was** isolated from

**with NaCl and other** a solar saltern with 313**‰ salinity. Extrapolation of the curve**

salts**^e, f^** suggests growth down to 0.767 a_w_ **(Park *et al*., 2007).**

**_____________________________________________________________________________________________________________________________________________________________________**

a. Germination, growth, and/or metabolic activity.

b. Values in bold represent water-activity minima for biotic activity of each species.

c. Growth and/or metabolic activity were determined empirically and stated water-activity values pertain to the temperature at which each study was carried out. Cultivation temperatures are given in brackets.

d. Those which differ from the empirical value (previous column) were derived by extrapolation. Stated water-activity values pertain to the temperature at which each study was carried out (see previous column in each case^c^) unless otherwise stated. na = not applicable.

e. The ionic composition of these media varied (see Park *et al*., 2006; 2007; and 2009).

f. These media were supersaturated (i.e. some precipitate was observed).

**Table S3.** Water-activity (a_w_) limits for biotic activity of xerotolerant Bacteria^a^ on high-sugar and other non-ionic substrates^b^.

**Bacterial species**^b^ **Substrate or cult- Lowest a_w_ at Lower a_w_ Notes and references**

**(strain[s]) ure medium, and which biotic limit for**

**added stressor(s) activity was biotic activity**

**empirically derived by**

**determined^c^  extrapolation^d^**

**_____________________________________________________________________________________________________________________________________________________________________**

**Actinobacteria**

*Micromonospora* **Yeast-Extract, Nitrate** **0.917** (10**°C) na Growth was observed over a range of glycerol concns. up to 2000**

*grisea* (JCM 3182) Agar supplemented mM **at 10°C**. **Spot-Test Assays indicated no cell division at 3000**

**with glycerol mM (see Stevenson and Hallsworth, 2014**)

*Micromonospora* sp. **Yeast-Extract, Nitrate** **0.920** (20**°C) na Growth was observed over a range of glycerol concns. up to 2000**

(JCM 3050) Agar supplemented mM **at 20°C**. **Spot-Test Assays indicated no cell division at 3000 with glycerol mM (see Stevenson and Hallsworth, 2014**)

***Mycobacterium*** Middlebrook 7H9 **0.800 (37°C) na** Assays were carried out using diverse stressors over a range of

***parascrofulaceum*** Broth supplemen- concns. and the most permissive solute was PEG 400 (see

**(LAIST_NPS017);** ted with PEG 400 **Santos *et al.,*** unpublished data)

***Mycobacterium smeg-***

***matis*** **(ATCC 10143)**

***Streptomyces albido-* Yeast-Extract, Nitrate** **0.895 (30°C) na Growth was observed over a range of glycerol concns. up to 3000**

***flavus* (JCM 4198) Agar supplemented mM at 30°C. Spot-Test Assays indicated no cell division at 4000**

**with glycerol mM (see Stevenson and Hallsworth, 2014**)

***Streptomyces*** *rectivio-* **Yeast-Extract, Nitrate**  **0.916** (4**°C) na Growth was observed over a range of glycerol concns. up to 2000**

*laceus* (JCM 9092) Agar supplemented mM **at 4°C**. **Spot-Test Assays indicated no cell division at 3000 with glycerol (see Stevenson and Hallsworth, 2014**)

**Bacilli**

***Bacillus cereus* (5B) Brain Heart Broth^e^ 0.921 (30°C) na Growth was observed over a range of glycerol concns. up to 3231**

**supplemented with mM (0.921 a_w_; Marshall *et al*., 1971)**

**glycerol**

***Bacillus megaterium* Brain Heart Broth^e^ 0.920 (30°C) na Growth was observed over a range of glycerol concns. up to 3266**

**(KM) supplemented with mM (0.920 a_w_; Marshall *et al*., 1971)**

**glycerol**

***Bacillus sphaericus* Brain Heart Broth^e^ 0.924 (30°C) na Growth was observed over a range of glycerol concns. up to 3123**

**(strain not specified) supplemented with mM (0.924 a_w_; Marshall *et al*., 1971)**

**glycerol**

***Bacillus subtilis* (W168; Brain Heart Broth^e^ 0.931 (30°C) na Growth was observed over a range of glycerol concns. up to 2865**

**ATCC 6051) supplemented with mM (0.931 a_w_; Marshall *et al*., 1971) glycerol**

***Lactococcus lactis*^f^** **Basal Milk Medium 0.930 (32°C**^f^**) 0.919 This bacterium grew at a considerable rate at 3367 mM glycerol**

**(strain not specified) supplemented with (0.930 a_w_) but was apparently inactive at 4398 mM glycerol**

**glycerol (0.910 a_w_; Troller and Stinson, 1981)**

***Lactobacillus plantarum* All Purpose Tween® 0.930 (30°C**^g^**) 0.924 This bacterium was able to multiple at a rate of 0.1 cells h^-1^ at**

**(strain not specified) 80 Broth supplem- 3367 mM glycerol (0.930 a_w_) but was inactive at 4398 mM**

**ented with glycerol glycerol (0.910 a_w_; Troller and Stinson, 1981)**

***Staphylococcus aureus* Casamino-Acid Yeast-0.860 (30°C) na Several strains of *S. aureus* (five out of the six assayed) were**

**(five strains) Extract, Casitone capable of growth at 3440 mM sucrose (0.860 a_w_; Scott, 1953) Broth supplemented**

**with sucrose**

***Staphylococcus epidermi-* Brain Heart Broth^e^ 0.901 (30°C) na Growth was observed over a range of glycerol concns. up to 3905**

***dis***^h^ **(strain not specified) supplemented with mM (0.901 a_w_; Marshall *et al*., 1971)**

**glycerol**

*Tetragenococcus halo-* Concentrated sugar- **0.841** (30**°C)** **na**  Two strains of *T. halophilus* **were capable of growth during a 24-d**

*philus* (T11; T15) beet extract containing **incubation period in a high-sucrose sugar-beet extract at 67°Bx**

**> 90% w/v sucrose**  **(0.841 a_w_;** Justé *et al*., 2008a; 2008b)

**Clostridia**

***Clostridium botulinum* Tryptone,Yeast Ext- 0.931 (30°C) na Growth was observed over a range of glycerol concns. up to 2819**

**(a type-A strain) ract, Glucose Broth mM (0.932 a_w_; Marshall *et al*., 1971)**

**supplemented with**

**glycerol**

***Sarcina* sp. (2b) Brain Heart Broth^e^ 0.918 (30°C) na Growth was observed over a range of glycerol concns. up to 3337**

**supplemented with mM (0.918 a_w_; Marshall *et al*., 1971)**

**glycerol**

**α-Proteobacteria**

*Asaia bogorensis* **Yeast-Extract, Urea,** **0.892 (28°C) 0.872 Growth rates were quantified over a range of glucose concns. up**

**(JCM 10569) Glucose Broth supp- to 2775 mM (0.892 a_w_). Extrapolation of the curve indicates**

**lemented with a theoretical growth limit of 3000 mM (0.872 a_w_; Jojima *et al.***

**glucose 2004)**

***Gluconacetobacter diaz-* Yeast-Extract, Urea, 0.892 (28°C) na Growth rates were quantified over a range of glucose concns. up**

***otrophicus* (DSM 5601) Dextrose Broth to 2775 mM (0.892 a_w_) at which growth ceased (Jojima *et al.*,**

**supplemented with 2004)**

**glucose**

***Pseudomonas putida* Luria-Bertani Broth 0.926 (30°C) 0.922 Growth rates were quantified over a range of glycerol concns. up**

**(**DSM 6125^i^) **supplemented with to 2000 mM (0.926 a_w_) and the theoretical limit, derived by extra-**

**glycerol polation, was 2120 mM (0.922 a_w_; see Cray *et al*. 2013a)**

***Saccharibacter floricola* Yeast-Extract, Urea, 0.892 (28°C) 0.849 Growth rates were quantified over a range of glucose concns. up**

**(**DSM 15669**) Dextrose Broth to 2775 mM (0.892 a_w_). Extrapolation of the curve indicates a**

**supplemented with theoretical growth limit of 3240 mM (0.849 a_w_; Jojima *et al.***

**glucose 2004)**

***Zymomonas mobilis* Yeast-Extract, Dext- 0.930 (30°C) <0.930 Growth rates were quantified over a range of glucose concns. up**

**(22 strains) rose Broth supplem- to 2220 mM (0.930 a_w_) at which 22 out of 40 strains were able**

**ented with glucose to grow after a lag phase of between 4 and 20 d (Swings and**

**De Ley, 1977)**

**γ-Proteobacteria**

***Halotalea alkalilenta* Yeast-Extract, Pep-0.913 (32°C) na Growth was observed over a range of glucose concns. up to 2498**

**(AW-7) tone Agar supplem- mM (0.913 w a_w_; Ntougias *et al*., 2007)**

**ented with glucose**

*Rosenbergiella nectarea* **Luria-Bertani Agar** **0.921** (28**°C) na Growth was observed over a range of sucrose** concns. up to 1753

(8N4; LMG 26121; supplemented with mM (Halpern *et al*., 2013) which is equivalent to 0.921 **a_w_** ^j^

DSM 24150) sucrose

__________________________________________________________________________________________________________________________

a. Growth and/or metabolic activity.

b. Only Bacteria are included due to a paucity of data for biotic activity of Archaea on sugar- or polyol-rich substrates at ≤ 0.937 **a_w_**. Values in bold represent water activity minima for biotic activity of each species.

c. Growth and/or metabolic activity were determined empirically and stated water-activity values pertain to the temperature at which each study was carried out. Cultivation temperatures are given in brackets.

d. Those which differ from the empirical value (previous column) were derived by extrapolation. Stated water-activity values pertain to the temperature at which each study was carried out (see previous column in each case^c^) unless otherwise stated^f^. na = not applicable.

e. This nutrient medium was made at one quarter standard strength (Marshall *et al*., 1971)

f. Formerly *Streptococcus lactis.*

g. Water-activity was determined at 25°C (Troller and Stinson, 1981).
h. Formerly *Staphylococcus albus.*

i. Commonly known as *P. putida* strain KT2440.

j. Water activity values were determined as described in ***Materials and methods***.

**Table S4.** Culture media used for determination of doubling times for halophilic Archaea and Bacteria^a^ at low water-activity (see Fig. 1a; Table 1).

**Added stressors/ substances (M)**

**Water activity NaCl MgCl_2_ MgSO_4_ Glycerol Ethylene**

**of medium^b^ glycol ___________________________________________________________________________________________________________________________________________________________________________________**

**0.803^c^ 3.50 0.399 0 0 0**

**0.775^d^ 5.10 0.0492 0 0.0130 0**

**0.717^e^  2.75 1.38 0 0 0**

**0.712^e^ 1.91 1.99 0 0 0**

**0.709^f^  3.33 2.00 0.0811 0 0**

**0.693^e^ 1.90 1.67 0 0 1.43**

**0.687^e^ 1.36 2.27 0 0 0**

**0.681^e^ 1.89 1.65 0 1.65 0**

**0.642^e^ 0.999 2.77 0 0 0**

**__________________________________________________________________________**

a. **Archaea *Halobacterium noricense* (DSM 15987**^T^**), *Halobacterium* sp. NRC-1, *Halococcus morrhuae* (NCIMB 787**^T^**), *Halococcus salifodinae* (DSM 13046), *Haloquadratum walsbyi* (DSM 16790), *Halorubrum saccharovorum* (NCIMB 2081**^T^**), and *Natrinema pallidum* (NCIMB 777**^T^**) and Bacteria *Pontibacillus* (strain AS2)** and *Salinicola* **(strain LC26; see Fig. 1a; Table 1).**

b. The 0.803 water-activity medium was used for cultivation of ***Hbt. noricense*, *Hbt.* sp. NRC-1, *Hcc. morrhuae*, *Hcc. salifodinae*, *Hrr. saccharovorum*, and *Nnm. pallidum* only**; the 0.775 water-activity medium for ***Pontibacillus* strain AS2 and** *Salinicola* **strain LC26 only;** and the 0.709 water-activity medium for *H. walsbyi* only (see Fig. 1a; Table 1). **Water activity values were determined at 37°C unless otherwise stated, as described in** *Materials and methods***.**

c. Modified **Yeast Extract, Casamino, Tri Sod**ium Citrate Broth A: yeast extract (10.0 g L^-1^; Difco, UK), casamino acids (7.5 g L^-1^), trisodium citrate (3.0 g L^-1^), K_2_SO_4_ (5.0 g L^-1^), FeCl_2_.4H_2_O (18.0 mg L^-1^), MnCl_2_.4H_2_O (0.18 mg L^-1^), and **NaCl and MgCl_2_** (see above); pH was adjusted to 7.6 with Tris-HCl.

d. Modified Casamino, Peptone, Starch Seawater (CPS-SW) Broth A: yeast extract (1 g L^-1^; Difco, UK), casamino acids (0.5 g L^-1^), bactopeptone (0.5 g L^-1^), soluble starch (0.5 g L^-1^), CaCl_2_.2H_2_O (73.5 mg L^-1^), Na_2_SO_4_ (71.0 mg L^-1^), KCl (37.3 mg L^-1^), and **NaCl, MgCl_2_** and glycerol (see above); pH was adjusted to <7 with PIPES (10 mM; Sass *et al*.*,* 2008). **Water activity was determined at 20°C, as described in** *Materials and methods***.** This medium was used for ***Pontibacillus* (strain AS2)** and *Salinicola* **(strain LC26) only; see Fig. 1a; Table 1.**

e. Modified **Yeast Extract, Casamino, Tri Sod**ium Citrate Broth B: yeast extract (10.0 g L^-1^; Difco, UK), casamino acids (7.5 g L^-1^,), trisodium citrate (3.0 g L^-1^), K_2_SO_4_ (1.0 g L^-1^), FeCl_2_.4H_2_O (36.0 mg L^-1^), MnCl_2_.4H_2_O (0.36 mg L^-1^), and **NaCl, MgCl_2_**, and/or glycerol or ethylene glycol (see above); pH was adjusted to 7.6 with Tris-HCl.

f. Modified Yeast Extract, Sodium Pyruvate Agar*:* agarose (10.0 g L^-1^), sodium pyruvate (1.0 g L^-1^), yeast extract (0.1 g L^-1^, Difco, UK), KCl (5.0 g L^-1^), NaNO_3_ (1.0 g L^-1^), CaCl_2_.2H_2_O (0.5 g L^-1^), NaHCO_3_ (0.25 g L^-1^), KH_2_PO_4_ (50.0 mg L^-1^), NH_4_Cl (30.0 mg L^-1^), and **NaCl, MgCl_2_**, MgSO_4_ (see above); pH was adjusted to 7.4 with Tris-HCl: for details see Bolhuis *et al*. (2004). This medium was supersaturated (i.e. some precipitate was observed) and was used for ***Hqr. walsbyi* (DSM 16790) only; see Fig. 1a; Table 1.**

**Table S5.** Culture media used to determine growth rates for *Halobacterium* strain 004.1 over a range of water-activity values (see Fig. 1d).

**Added stressors/ salts (M)**

**Water activity NaCl MgCl_2_ Na_2_SO_4_ KCl MgBr_2_**

**of medium^a^**

**__________________________________________________________________________**

**0.980 0.362 0.0466 0.0248 0.0869 0.000400**

**.**

**0.961 0.724 0.0936 0.0495 0.0174 0.000821**

**0.940 1.089 0.140 0.0743 0.0260 0.00111**

**0.917 1.61 0.187 0.0990 0.0347 0.00148**

**0.892 1.45 0.233 0.124 0.0435 0.00185**

**0.864 2.17 0.281 0.149 0.0521 0.00246**

**0.833 2.53 0.328 0.174 0.0608 0.00287**

**0.800 2.90 0.374 0.198 0.0697 0.00329**

**0.765 3.26 0.421 0.223 0.0782 0.00369**

**0.728 3.62 0.466 0.248 0.0869 0.004107**

**__________________________________________________________________________**

a. Modified Casamino, Peptone, Starch Seawater (CPS-SW) Broth B: CaCl_2_ (0.774 g L^-1^); Stock Solution A^b^ (79.7 L^-1^), Stock Solution B^c^ (20.3 L^-1^), and **NaCl, MgCl_2_, Na_2_SO_4_, KCl, and MgBr_2_** (see above); buffered with Tris-HCl to a pH of 7.6. The concentrations given for added stressors and CaCl_2_ are pertinent to the medium in which the cells were cultured. *Halobacterium* strain 004.1 was grown in 10 ml aliquots of the final medium incubated at 37°C; water activity values were determined empirically as described in *Materials and methods*.

b. Stock Solution A: Element Solution SL10 (20.0 ml L^-1^; Widdel *et al*., 1983), glycerol (1.0 ml L^-1^), 0.5 M NH_4_Cl (1.0 ml L^-1^), 0.1 M KH_2_PO_4_ (1.0 ml L^-1^), 0.4 M SrCl_2_.6H_2_O (0.75 ml L^-1^), 0.07 M NaF (0.71 ml L^-1^), 0.4 M H_3_BO_3_ (0.6 ml L^-1^) and soluble starch (0.5 g L^-1^).

c. Stock Solution B: casamino acids (50.0 g L^-1^), bactopeptone (50.0 g L^-1^), vitamin solution (5x Balch; 28.0 ml L^-1^). Solution B was filter sterilized separately.

**Table S6.** Culture media used to determine growth rates for *Salinibacter ruber* over a range of water-activity values (see Fig. 1e).

**Added salts (M) or seawater (ml L^-1^)**

**Water activity NaCl MgCl_2_ MgSO_4_  Water from the**

**of medium Dead Sea^a^ _______________________________________________________________________**

***Supplemented with MgCl_2_+NaCl or NaCl+water from the Dead Sea^b^***

0.840^c^ 3.34 0.0801 0.101 0

0.812^c^ 2.80 0.0673 0.0852 160

0.801^c^ 2.27 0.0545 0.0689 320

0.790^c^ 1.74 0.0417 0.0527 480

0.777^c^ 1.20 0.0289 0.0365 640

***Primary added stressor = NaCl^d^***

**0.903^e^ 2.00 0.146 0.121 0**

**0.844^f^ 3.34** 0.0801 **0.101 0**

**0.836^g^  3.34 0.243 0.140 0**

**0.752^h^ 5.86^i^ 0.170 0.201 0**

_________________________________________________________________________________________________________________________________________________________________________________

a. Water from the Dead Sea contains ~35% w/v total dissolved salts, predominantly MgCl_2_ (2.05 M), NaCl (1.48 M) and CaCl_2_ (0.5 M); these data were obtained from the Geological Survey of Israel (see also Bodaker *et al*., 2010).

b. See Fig. 1e (yellow line).

c. Modified Yeast Extract, Salts Broth: yeast extract (1.0 g L^-1^, Beckton, Dickinson and Company, Sparks, USA), KCl (5.0 g L^-1^), CaCl_2_.2H_2_O (1.25 g L^-1^), NaBr (0.625 g L^-1^), NaHCO_3_ (0.25 g L^-1^), and **NaCl, MgCl_2_, MgSO_4_** and/or water from the Dead Sea (see above); pH was adjusted to 7 with NaOH. Water activity was determined at 35°C, as described in *Materials and methods*.

d. See Fig 1e (black line).

e. 15% w/v SW Medium B: yeast extract (1.0 g L^-1^, Difco), KCl (5.0 g L^-1^), CaCl_2_.2H_2_O (1.25 g L^-1^), NaBr (0.625 g L^-1^), NaHCO_3_ (0.25 g L^-1^), and **NaCl, MgCl_2_, and MgSO_4_** (see above); pH was adjusted to 7 with NaOH. **Water activity was determined at 35°C, as described in** *Materials and methods* **(see also Antón *et al*., 2002).**

f. 25% w/v SW Medium B: yeast extract (1.0 g L^-1^, Difco), KCl (5.0 g L^-1^), CaCl_2_.2H_2_O (1.25 g L^-1^), NaBr (0.625 g L^-1^), NaHCO_3_ (0.25 g L^-1^), and **NaCl, MgCl_2_, and MgSO_4_** (see above); pH was adjusted to 7 with NaOH. **Water activity was determined at 35°C, as described in** *Materials and methods* **(see also Antón *et al*., 2002; Sher *et al*., 2004).**

g. SW25% Medium: yeast extract (2.0 g L^-1^, Difco), KCl (5.0 g L^-1^), CaCl_2_.2H_2_O (1.25 g L^-1^), NaBr (0.625 g L^-1^), NaHCO_3_ (0.25 g L^-1^); pH was adjusted to 7 with NaOH. **Water activity was determined at 35°C, as described in** *Materials and methods* **(see also Pe**ñ**a *et al*., 2010).**

**h.** SW25% Medium plus NaCl to saturation: yeast extract (2.0 g L^-1^, Difco), KCl (5.0 g L^-1^), CaCl_2_.2H_2_O (1.25 g L^-1^), NaBr (0.625 g L^-1^), NaHCO_3_ (0.25 g L^-1^), and **NaCl, MgCl_2_, and MgSO_4_** (see above); pH was adjusted to 7 with NaOH. **Water activity was determined at 35°C, as described in** *Materials and methods* **(see also Pe**ñ**a *et al*., 2010).**

i. This medium was supersaturated (i.e. some precipitate was observed).

**Table S7.** Culture media used to determine growth rates for *Salisaeta longa* over a range of water-activity values (see Fig. 1f).

**Added salts (M) or seawater (ml L^-1^)**

**Water activity NaCl MgCl_2_ Water from the**

**of medium Dead Sea^a^ _______________________________________________________________**

***Supplemented with water from the Dead Sea***^b^

0.926^c^ 0 0 160

0.887^c^ 0 0 320

0.826^c^  0 0 480

0.792^c^  0 0 640

***Primary added stressor = NaCl^d^***

0.887^e^ 2.10 0.00984 0

0.876^e^ 2.48 0.00984 0

0.864^e^ 2.87 0.00984 0

0.850^e^ 3.25 0.00984 0

0.827^e^ 3.76 0.00984 0

0.805^e^ 4.28 0.00984 0

_____________________________________________________________________

a. Water from the Dead Sea contains ~35% w/v total dissolved salts, predominantly MgCl_2_ (2.05 M), NaCl (1.48 M) and CaCl_2_ (0.5 M); these data were obtained from the Geological Survey of Israel (see also Bodaker *et al*., 2010).

b. See Fig. 1f (yellow line).

c. Modified Yeast Extract, Casamino, Starch Broth A: soluble starch (2.0 g L^-1^, Mallinckrodt Baker, Deventer, the Netherlands), yeast extract (1.0 g L^-1^, Beckton, Dickinson and Company, Sparks, USA), casamino acids (1.0 g L^-1^, Beckton, Dickinson and Company, Sparks, USA), and water from the Dead Sea (see above); pH was adjusted to 7 with NaOH. Water activity was determined at 35°C, as described in *Materials and methods*.

d. See Fig. 1f (black line).

e. Modified Yeast Extract, Casamino, Starch Broth B: soluble starch (2.0 g L^-1^, Mallinckrodt Baker, Deventer, the Netherlands), yeast extract (1.0 g L^-1^, Beckton, Dickinson and Company, Sparks, USA), casamino acids (1.0 g L^-1^, Beckton, Dickinson and Company, Sparks, USA), K_2_SO_4_ (5.0 g L^-1^), CaCl_2_.2H_2_O (0.1 g L^-1^), and NaCl, and MgCl_2_ (see above); pH adjusted to 7 with NaOH. Water activity was determined at 35°C, as described in *Materials and methods*.

**Table S8.** Culture media used to determine growth rates for *Aspergillus penicillioides* and *Xeromyces bisporus* over a range of water-activity values (see Figs. 3 and 4)**^a^.**

**Added stressors/ substances (M)**

**Medium water Glycerol Sucrose Glucose Fructose Ethylene Betaine Proline MgCl_2_ NaCl KCl**

**activity (*pH*)^a^ glycol __________________________________________________________________________________________________________**

0.934 (*5.77*) 0 0 0 0 0 1.71 0 0 0 0

0.928 (*6.50*) 0 0 0 0 2.00 0 0 0 0 0

0.927 (*6.00*) 0 0 0 0 0 0 2.61 0 0 0

0.915 (*5.50*) 0 0 0 0 0 0 0 0.70 0 0

0.907 (*5.50*) 0 0 0 0 0 0 0 1.0 0 0

0.904 (*5.64*) 0 0 0 0 2.56 0 0 0 0 0

0.890 (*5.00*) 0 0 0 0 0 0 0 0 0 0

0.880 (*6.00*) 0 0 0 0 0 0 3.47 0 0 0

0.859 (*5.31*) 0 2.19 0 0 0 0 0 0 0 0

0.842 (*6.50*) 0 0 2.04 0 0 0 0 0 0 0

0.828 (*5.87*) 4.34 0 0 0 0 0 0 0 0 0

0.822 (*6.00*) 4.34 0 0 0 0 0 0 0 0 0

0.814 (*5.80*) 0 0 0 0 0 0 4.34 0 0 0

0.810 (*4.09*)^b^ 5.67 0 0 0 0 0 0 0 0 0

0.805 (*4.17*)^c^ 5.61 0 0 0 0 0 0 0 0 0

0.804 (*5.79*) 5.64 0 0 0 0 0 0 0 0 0

0.801 (*6.97*)^b^ 5.61 0 0 0 0 0 0 0 0 0

0.799 (*5.70*) 5.64 0 0 0 0 0 0 0 0 0

0.798 (*6.94*)^d^ 5.58 0 0 0 0 0 0 0 0 0

0.795i^e^ (*6.96*) 5.64 0 0 0 0 0 0 0 0 0

0.795ii^e^ (*5.68*)^b^ 5.61 0 0 0 0 0 0 0 0 0

0.788 (*4.32*) 0 0 0 4.66 0 0 0 0 0 0

0.779 (*5.89*) 3.31 0 0 0 0 2.06 0 0 0 0

0.776 (*5.58*) 4.76 0 0 0 0 0 0 0 1.11 0.13

0.773 (*5.71*) 1.99 0 0 0 0 2.89 0 0 0 0

0.772 (*5.72*) 6.26 0 0 0 0 0 0 0 0 0

0.770 (*7.00*) 6.26 0 0 0 0 0 0 0 0 0

0.763 (*6.97*)^b^ 6.22 0 0 0 0 0 0 0 0 0

0.752 (*5.71*) 3.86 0 0 0 0 1.20 0 0 0.86 0.13

0.750 (*5.74*) 6.29 0 0 0 0 0 0 0 0 0

0.748 (*5.85*)^f^ 6.29 0 0 0 0 0 0 0 0 0

0.744 (*5.75*) 0 0 1.11 3.89 0 0 0 0 0 0

0.741 (*5.68*) 0 0 1.11 3.89 0 0 0 0 0 0

0.740 (*4.05*) 6.86 0 0 0 0 0 0 0 0 0

0.735 (*6.96*) 6.86 0 0 0 0 0 0 0 0 0

0.731 (*5.71*)^b^  6.22 0 0 0 0 0 0 0 0 0

0.723 (*4.16*)^b^ 6.82 0 0 0 0 0 0 0 0 0

0.718 (*5.48*) 0 0 1.11 3.61 0 0 0 0 0 0

0.716 (*5.69*)^b^ 6.82 0 0 0 0 0 0 0 0 0

0.714i^e^ (*5.54*) 0 0 1.11 4.16 0 0 0 0 0 0

0.714ii^e^ (5.75) 6.84 0 0 0 0 0 0 0 0 0

0.707 (*4.85*) 3.26 1.02 0 0 0 0 0 0 1.20 0.13

0.706 (*5.39*) 2.72 0 1.11 1.55 0 0 0 0 0 0

0.702i^e^ (*5.58*) 4.14 0 0 0 0 1.20 0 0 0.86 0.13

0.702ii^e^ (*5.64*) 7.06 0 0 0 0 0 0 0 0 0

0.701 (*5.38*) 3.80 0 1.11 1.11 0 0 0 3.47 0 0

0.695 (*5.48*) 3.26 0 1.11 1.55 0 0 0 0 0 0

0.690 (*5.39*) 4.88 1.17 0 0 0 0 0 0 0 0

0.681 (*5.32*) 3.80 0 0.56 1.78 0 0 0 0 0 0

0.670 (*5.70*) 5.97 0.73 0 0 0 0 0 0 0 0

0.667 (*5.73*) 3.91 0.73 0 0 0 0 0 0 1.20 0.13

0.665 (*4.19*) 7.44 0 0 0 0 0 0 0 0 0

0.656 (*5.34*) 4.72 0.73 0 0 0 0 0 0 1.20 0.13

0.655i^e^ (*5.51*)^b^ 0 0 0.55 4.44 0 0 0 0 0 0

0.655ii^e^ (*4.03)* 7.60 0 0 0 0 0 0 0 0 0

0.654 (*5.31*) 5.43 1.02 0 0 0 0 0 0 0 0

0.653 (*5.68*) 7.60 0 0 0 0 0 0 0 0 0

0.647 (*5.50*) 6.19 0 0 0 0 0 0 0 1.20 0.13

0.640 (*5.69*) 4.34 0 0.55 1.78 0 0 0 0 0 0

0.639 (*5.45*) 5.23 0.73 0 0 0 0 0 0 1.20 0.13

0.634 (*5.08*)^g^  6.45 0.73 0 0 0 0 0 0 0 0

0.631 (*4.84*) 4.87 0 0.55 1.78 0 0 0 0 0 0 **___________________________________________________________________________________________________________**

**a. Modified Malt Extract, Yeast Extract, Phosphate Agar (MYPiA): malt extract (1% w/v; Oxoid, UK), yeast extract (1% w/v; Oxoid, UK), K_2_HPO_4_ (0.1% w/v), Agar (1.5% w/v; Acroid, USA), and added stressors (see above); buffered with citric acid or Na_2_HPO_4_ and incubated at 30°C unless otherwise specified (*See also* Williams and Hallsworth, 2009). Water activity values were determined at the same temperature at which each medium was incubated (see *Materials and methods*); chaotropic or kosmotropic activities of media with differing solute composition varies (see Hallsworth *et al*., 2003a; 2007; Williams and Hallsworth, 2009; Chin *et al*., 2010; Cray *et al*., 2013a; 2013b).**

**b. Incubated at 37.5°C.**

**c. Incubated at 22.5°C.**

**d. Incubated at 15°C.**

**e. Whereas some media with a differing solute composition had an identical water activity value, their respective chaotropic or kosmotropic activities differ (see Williams and Hallsworth, 2009).**

**f. Concentrations of yeast extract, malt extract and KH_2_PO_4_ were 10-fold more dilute in this medium.**

**g. Concentrations of yeast extract, malt extract and KH_2_PO_4_ were 10-fold more concentrated in this medium.**

**Supporting references**

Andrews S, Pitt JI. (1987). Further studies on the water relations of xerophilic fungi, including some halophiles. *J Gen Microbiol* 133: 233–238.

Antón J, Oren A, Benlloch S, Rodriguez-Valera F, Amann R, Rossello-Mora R. (2002) *Salinibacter ruber* gen. nov., a novel, extremely halophilic member of the bacteria from saltern crystallizer ponds. *Int J Syst Evol Microbiol* 52: 485–491.

Bolhuis H, Poele EM, Rodriguez-Valera F. (2004). Isolation and cultivation of Walsby’s square archaeon. *Environ Microbiol* 6: 1287–1291.

Brown AD. (1990). *Microbial Water Stress Physiology*. *Principles and Perspectives.* John Wiley and Sons, Chichester, United Kingdom.

Butinar L, Zalar P, Frisvad JC, Gunde-Cimerman N. (2005). The genus *Eurotium* – members of indigenous fungal community in hypersaline waters of salterns. *FEMS Microbiol Ecol* 51: 155–166.

Cayol J–L, Ollivier B, Patel BKC, Ageron E, Grimont PAD, Prensier G *et al*. (1995). *Haloanaerobium lacusroseus* sp. nov., an extremely halophilic fermentative bacterium from the sediments of a hypersaline lake. *Int J Syst Bacteriol* 45: 790–797.

Chin JP, Megaw J, Magill CL, Nowotarski K, Williams JP, Bhaganna P *et al.* (2010). Solutes determine the temperature windows for microbial survival and growth. *Proc Natl Acad Sci* 107: 7835–7840.

Cifuentes AS, González MA, Inostroza I, Aguilera A. (2001). Reappraisal of physiological attributes of nine strains of *Dunaliella* (Chlorophyceae): growth and pigment content across a salinity gradient. *J Phycol* 37: 334–344.

Cray JA, Bell ANW, Bhaganna P, Mswaka AY, Timson DJ, Hallsworth JE. (2013a). The biology of habitat dominance; can microbes behave as weeds? *Microb Biotechnol* 6: 453–492.

Cray JA, Russell JT, Timson DJ, Singhal RS, Hallsworth JE. (2013b). A universal measure of chaotropicity and kosmotropicity. *Environ Microbiol* 15: 287–296.

Daffonchio D, Borin S, Brusa T, Brusetti L, van der Wielen PWJJ, Bolhuis H *et al*. (2006). Stratified prokaryote network in the oxic-anoxic transition of a deep-sea halocline. *Nature* 440: 203–207.

Deole R, Challacombe J, Ralford DW, Hoff WD. (2013). An extremely halophilic proteobacterium combines a highly acidic proteome with a low cytoplasmic potassium content. *J Biol Chem* 288: 581–588.

Gock MA, Hocking AD, Pitt JI, Poulos PG. (2003). Influence of temperature, water activity and pH on growth of some xerophilic fungi. *Int J Food Microbiol* 81:11–19.

Gunde-Cimerman N, Zalar P, de Hoog S, Pleminitaš A. (2000). Hypersaline waters in salterns − natural ecological niches for halophilic black yeasts. *FEMS Microb Ecol* 32: 235–240.

Hallsworth JE, Heim S, Timmis KN. (2003a). Chaotropic solutes cause water stress in *Pseudomonas putida*. *Environ Microbiol* 5: 1270–1280.

Hallsworth JE, Yakimov MM, Golyshin PN, Gillion JLM, D'Auria G, Alves FL *et al*. (2007). Limits of life in MgCl2-containing environments: chaotropicity defines the window. *Environ Microbiol* 9: 803–813.

Halpern M, Fridman S, Atamna-Ismaeel N, Izhaki I. (2013). *Rosenbergiella nectarea* gen nov. sp. nov., in the family *Enterobacteriaceae*, isolated from floral nectar. *Int J Syst Evol Microbiol* 63: 4259–4265.

Javor BJ, Requadt C, Stoeckenius W. (1982). Box-shaped halophilic bacteria. *J Bacteriol* 151: 1532–1542.

Javor BJ. (1984). Growth potential of halophilic bacteria isolated from solar salt environments: carbon sources and salt requirements. *Appl Environ Microbiol* 48: 352–360.

Javor BJ. (1989). *Hypersaline environments: microbiology and biogeochemistry*. Springer-Verlag, Berlin.

Jojima Y, Mihara Y, Suzuki S, Yokozeki K, Yamanaka S, Fudou R. (2004). *Saccharibacter floricola* gen. nov., sp. nov., a novel osmophilic acetic acid bacterium isolated from pollen. *Int J Syst Evol Micr* 54: 2263–2267.

Justé A, Lievens B, Klingeberg M, Michiels CW, Marsh TL, Willems KA. (2008a). Predominance of *Tetragenococcus halophilus* as the cause of sugar thick juice degradation. *Food Microbiology* 25: 413–421.

Justé A, Lievens B, Frans I, Marsh TL, Klingeberg M, Michiels CW, *et al*. (2008b). Genetic and physiological diversity of *Tetragenococcus halophilus* strains isolated from sugar- and salt-rich environments. *Microbiology* 154: 2600–2610.

Leong SL, Pettersson OV, Rice T, Hocking AD, Schnürer J. (2011). The extreme xerophilic mould *Xeromyces* *bisporus* – growth and competition at various water activities. *Int J Food Microbiol* 145: 57–63.

Marshall BJ, Ohye DF, Christian JHB. (1971). Tolerance of bacteria to high concentrations of NaCl and glycerol in the growth medium. *Appl Microbiol* 21: 363–364.

Ntougias S, Zervakis GI, Fasseas C. (2007). *Halotalea alkalilenta* gen. nov., sp. nov., a novel osmotolerant and alkalitolerant bacterium from alkaline olive mill wastes, and emended description of the family *Halomonadaceae* Franzmann *et al*. 1989, emend. Dobson and Franzmann 1996. *Int J Syst Evol Microbiol* 57: 1975–1983.

Park JS, Cho BC, Simpson AGB. (2006). *Halocafeteria seosinensis* gen. et sp. nov. (Bicosoecida), a halophilic bacterivorous nanoflagellate isolated from a solar saltern. *Extremophiles* 10: 493–504.

Park JS, Simpson AG, Lee WJ, Cho BC. (2007). Ultrastructure and phylogenetic placement within Heterolobosea of the previously unclassified, extremely halophilic heterotrophic flagellate *Pleurostomum flabellatum* (Ruinen 1938). *Protist* 159: 397–413.

Park JS, Simpson AG, Brown S, Cho BC. (2009). Ultrastructure and molecular phylogeny of two heterolobosean amoebae, *Euplaesiobystra hypersalinica* gen. et sp. nov. and *Tulamoeba peronaphora* gen. et sp. nov., isolated from an extremely hypersaline habitat. *Protist* 160: 265–283.

Payne JI, Sehgal SN, Gibbons NE. (1960). Immersion refractometry of some halophilic bacteria. *Can J Microbiol* 6: 9–15.

Peña A, Teeling H, Huerta-Cepas J, Santos F, Yarza P, Brito-Echeverría J *et al*. (2010). Fine-scale evolution: genomic, phenotypic and ecological differentiation in two coexisting *Salinibacter ruber* strains. *ISME J* 4: 882–895.

Pitt JI, Christian JHB. (1968). Water relations of xerophilic fungi isolated from prunes. *Appl Environ Microbiol* 16: 1853–1858.

Pitt JI, Hocking AD. (1977). Influence of solute and hydrogen ion concentration on the water relations of some xerophilic fungi. *J Gen Microbiol* 101: 35–40.

**Pitt JI, Hocking AD. (2009). *Aspergillus.* In *Fungi and Food Spoilage* (3rd edition). Springer, New York, USA.**

Sass AM, McKew BA, Sass H, Fichtel J, Timmis KN, McGenity TJ. (2008). Diversity of *Bacillus*-like organisms isolated from deep-sea hypersaline anoxic sediments. *Saline Systems* 4: 8.

Scott WJ. (1953) Water relations of *Staphylococcus aureus* at 30°C. *Aust J Biol Sci* 6: 549–564.

Sher J, Elevi R, Mana L, Oren A. (2004). Glycerol metabolism in the extremely halophilic bacterium *Salinibacter ruber*. *FEMS Microbiol Lett* 19: 211–215.

Snow D. (1949). The germination of mould spores at controlled humidities. *Ann Appl Biol* 36: 1–13.

Stevenson A, Hallsworth JE. (2014). Water and temperature relations of soil Actinobacteria. *Environ Microbiol Rep* In press.

Swings J, De Ley J. (1977). The biology of *Zymomonas*. *Bacteriol Rev* 41: 1-46.

Troller JA, Stinson JV. (1981). Moisture requirements for growth and metabolite production by lactic acid bacteria. *Appl Environ Microbiol* 42: 682-687.

**von Schelhorn M. (1950).** Untersuchungen uber den verderb wasserarmer lebensmittel durch osmophile mikroorganismen. II. grenzkonzentrationen fur den osmophilen schimmelpilz *Aspergillus glaucus* in abhangigkeit vom pH wert des substrates. *Z. Lebensm.* *Untersuch* *Forsch* 91: 338–342.

Wainø M, Tindall BJ, Ingvorsen K. (2000). *Halorhabdus utahensis* gen. nov., sp. nov., an aerobic, extremely halophilic member of the Archaea from Great Salt Lake, Utah. *Int J Syst Evol Micr* 50: 183–190.

Wheeler KA, Hocking AD, Pitt JI. (1988). Influence of temperature on the water relations of *Polypaecilium pisce* and *Basipetospora halophila,* two halophilic fungi. *J Gen Microbiol* 134: 2255–2260.

Winston PW, Bates PS. (1960). Saturated salt solutions for the control of humidity in biological research. *Ecology* 41: 232–237.

Williams JP, Hallsworth JE. (2009). Limits of life in hostile environments; no limits to biosphere function? *Environ Microbiol* 11: 3292–3308.

Yoshida M, Matsubara K, Kudo T, Horikoshi K. (1991). *Actinopolyspora mortivallis* sp. nov. a moderately halophilic actinomycete. *Int J Syst Bacteriol* 41: 15–20.

Zalar P, de Hoog GS., Schroers HJ, Crous PW, Groenwald JZ, Gunde-Cimerman N. (2007). Phylogeny and ecology of the ubiquitous saprobe *Cladosporium sphaerospermum*. *Studies in Mycology* 58: 157–183.
